# Predictive Performance of Neuron-Specific Enolase (NSE) for Survival after Resuscitation from Cardiac Arrest: A Systematic Review and Meta-Analysis

**DOI:** 10.3390/jcm12247655

**Published:** 2023-12-13

**Authors:** Krzysztof Kurek, Damian Swieczkowski, Michal Pruc, Monika Tomaszewska, Wieslaw Jerzy Cubala, Lukasz Szarpak

**Affiliations:** 1 Department of Clinical Research and Development, LUXMED Group, 02-676 Warsaw, Poland; 2 Department of Toxicology, Faculty of Pharmacy, Medical University of Gdansk, 80-210 Gdansk, Poland; 3 Research Unit, Polish Society of Disaster Medicine, 05-806 Warsaw, Poland; 4 Department of Public Health, International Academy of Ecology and Medicine, 02000 Kyiv, Ukraine; 5 Department of Psychiatry, Medical University of Gdansk, 80-210 Gdansk, Poland; cubala@gumed.edu.pl; 6 Institute of Outcomes Research, Maria Sklodowska-Curie Medical Academy, 03-411 Warsaw, Poland; 7 Henry JN Taub Department of Emergency Medicine, Baylor College of Medicine, Houston, TX 77030, USA; 8 Research Unit, Maria Sklodowska-Curie Bialystok Oncology Center, 15-027 Bialystok, Poland

**Keywords:** neuron-specific enolase, NSE, cardiopulmonary resuscitation, cardiac arrest, biomarker, biological marker, prognosis, survival, evidence-based medicine, meta-analysis

## Abstract

The prediction of outcomes following cardiac arrest continues to provide significant difficulties. A preferred strategy involves adopting a multimodal approach, which encompasses the careful evaluation of the biomarker neuron-specific enolase (NSE). This systematic review and meta-analysis aimed to gather and summarize new and existing evidence on the prediction effect of neuron-specific enolase for survival to hospital discharge among adult patients with cardiac arrest. We searched PubMed Central, Scopus, EMBASE databases, and the Cochrane Library without language restrictions from their inceptions until 30 October 2023 and checked the reference lists of the included studies. Pooled results were reported as standardized mean differences (SMDs) and were presented with corresponding 95% confidence intervals (CIs). The primary outcome was survival to hospital discharge (SHD). Eighty-six articles with 10,845 participants were included. NSE showed a notable degree of specificity in its ability to predict mortality as well as neurological status among individuals who experienced cardiac arrest (*p* < 0.05). This study demonstrates the ability to predict fatality rates and neurological outcomes, both during the time of admission and at various time intervals after cardiac arrest. The use of NSE in a multimodal neuroprognostication algorithm has promise in improving the accuracy of prognoses for persons who have undergone cardiac arrest.

## 1. Introduction

In the context of medical prognoses following sudden cardiac arrest (SCA), outcomes are often unfavorable, particularly when cerebral damage results from acute oxygen deprivation [1,2]. Acute oxygen deprivation is not the only cause for brain injury, but also the lack of perfusion due to a sudden drop in cardiac output. Prognostic factors, such as the presence of shockable cardiac rhythms or the occurrence of SCA in the presence of witnesses, can be readily identified [3]. Advanced age, especially that over 80 years, exerts an additional detrimental influence on patient prognoses [4], and given our aging population, the prevalence of such cases is increasing. SCA survivors form a diverse group, with those maintaining neurological functions facing challenges in post-hospital care, rehabilitation, and preventing recurrent cardiac events through interventions like cardioverter–defibrillators or percutaneous coronary interventions [5].

Cerebral injuries from SCA often lead to significant neurological deficits, including the need for prolonged mechanical ventilation. Patients may also experience long-term immobilization, speech disorders, and cognitive impairments, all contributing to a bleaker prognosis and higher mortality rates after SCA [6]. Prolonged cerebral hypoxia exacerbates intracranial pressure, especially in the presence of post-SCA brain injuries [6]. Secondary brain injuries occur as a result of many mechanisms, including as a result of an imbalance of ions in the intra- and extracellular space in particular calcium ions. The activation of a number of enzymes dependent on calcium ions leads to further damage due to the destruction of cell organelles and an increase in oxidative stress. Secondarily, the immune system is activated, and the blood–brain barrier is breached [7]. Clinicians grapple with differentiating between patients with promising prospects for brain damage recovery and those with limited improvement potential, a complex task. Neuroimaging advancements offer enhanced cerebral visualization but fall short of providing definitive prognostic insights, prompting research into neurobiomarkers [8,9].

One key neurobiomarker in this context is neuron-specific enolase (NSE), primarily found in neurons and neuroendocrine cells. Elevated NSE levels following brain injury result from various mechanisms, including damage to nerve cells leading to NSE release into the extracellular space. This release stems not only from necrosis but also from apoptosis due to the indirect consequences of brain injury. Additionally, damage to the blood–brain barrier can significantly raise NSE concentrations [10]. Despite ongoing debates regarding its clinical utility, it is crucial to note that even minor hemolysis in specimens can substantially inflate NSE test results [11,12]. Modern laboratory techniques have been developed to minimize the risk of hemolysis-related false positives.

Numerous publications have suggested that incorporating NSE levels into existing prognostic tools can optimize their predictive capabilities. Luescher et al. found that measuring NSE levels on the third day after a patient admission to an intensive care unit (ICU) improved clinical risk scores (the out-of-hospital cardiac arrest score and the Cardiac Arrest Hospital Prognosis Score—CAHP) for predicting the outcomes of cardiac arrest patients in terms of neurological outcomes and in-hospital mortality [13]. Recent research has found specific concentration thresholds that indicate brain damage. For example, concentrations above 100 g/L are linked to poor neurological outcomes and a high level of specificity, while concentrations below 17 g/L show that severe encephalopathy is not present. The authors also highlighted potential confounding factors, such as extracorporeal membrane oxygenation (ECMO), malignancies, or blood transfusions, which could affect NSE marker accuracy. Importantly, these factors elevated the risk of hemolysis, interfering with accurate NSE determination [14]. Nevertheless, repeated measurements indicating an increase in NSE concentration values between 48 and 72 h after cardiac arrest may have been an element of an algorithm predicting poor neurological outcomes [15]. The proposed other prognostic algorithm could also include a combination of neurofilament light (NfL) and NSE by measuring NfL at 24, 48, and 72 h after cardiac arrest, and NSE at 72 h [16]. Previously published meta-analyses did not include all newly published articles, so there is a scientific justification for a new meta-analysis [17,18,19].

Considering these factors, this meta-analysis aimed to evaluate the utility of the NSE neurobiomarker in predicting survival to hospital discharge among SCA patients.

## 2. Materials and Methods

This systematic review was conducted in line with the Preferred Reporting Items for Systematic Reviews and Meta-Analyses (PRISMA) [20] and the Meta-Analysis of Observational Studies in Epidemiology (MOOSE) [21] guidelines. The protocol was developed a priori and accepted by all authors, and no protocol changes were made during the study. The review protocol was prospectively submitted and registered in the PROSPERO database (registration number: CRD42023468523). Due to the character of this study, the ethics committee portion was not applicable.

### 2.1. Literature Search and Selection

A literature search was performed in the PubMed Central, Scopus, and EMBASE databases, as well as the Cochrane Central Register of Controlled Trials, and included all articles from inception to the date of abstract extraction on 30 October 2023. The phrases we used for the literature search were as follows: "neuron-specific enolase" OR "NSE" AND “cardiac arrest” OR “out-of-hospital cardiac arrest” OR “OHCA” OR “In-hospital cardiac arrest” OR “heart arrest” OR “cardiopulmonary resuscitation” OR “CPR” OR “sudden cardiac death” (Table S1).

We performed more surveillance searches using the “related articles” feature, and we also performed a thorough search of unpublished literature about how NSE infection affects survival after cardiac arrest. This search encompassed the reference lists of all the included studies and existing traditional systematic reviews, as well as gray literature sources such as Google Scholar. The elimination of duplicate findings was undertaken. Two writers, K.K. and M.P., conducted separate assessments to determine the relevance of the remaining works. The third researcher (L.S.) checked the list of pertinent publications.

The application of inclusion and exclusion criteria was extended to encompass the entirety of the remaining articles’ content. The filters employed encompassed many criteria, namely the exclusion of human studies involving individuals aged 18 or older who had cardiac arrests, the requirement for studies to be conducted in the English language, the inclusion of both prospective and retrospective observational studies, and the condition that the studies were published in peer-reviewed journals.

The following details regarding the search strategy, in the broader context of the review question and selection of papers, needs to be clarified: P (population): patients suffering from cardiac arrest (out-of-hospital or in-hospital-cardiac arrest); I (intervention): the determination of neuron-specific enolase (NSE); OHCA: treated with cardiopulmonary resuscitation; C (comparator): not applicable; O (outcomes): survival of OHCA/IHCA to hospital discharge; S (study design): observational studies (inc. cross-sectional studies), non-randomized and randomized clinical trials (if applicable); T (time frame): index time: (i) 6 h, (ii) 12 h, (iii) 24 h, (iv) 48 h, and (v) 72 h after OHCA. 

The purpose of the meta-analysis was not to compare NSE to other biomarkers; hence, no comparator was indicated.

### 2.2. Data Extraction and Quality Assessment

K.K. and M.P. independently extracted the data from each of the included studies and entered them into a thorough spreadsheet, which a third reviewer (L.S. or M.T.) independently verified. The extracted data comprised the baseline and methodological features of the studies, that is, the first author’s name, the country in which the study was conducted, study design, total participants, study population, age, sex, NSE levels, in-hospital survival, and the mortality rate.

The assessment of the risk of bias in each study was independently carried out by two reviewers using standardized tools. The Newcastle–Ottawa Scale (NOS) [22] was used by the authors to independently assess (K.K. and M.T.) the risk of bias in each study. Quality rating disagreements were resolved by discussion among all authors. We utilized the NOS to assess the quality of each observational study, which included judgments on the selection of study subjects, the comparability between study groups, as well as the outcomes of each study. The total scores that could be obtained using this tool were 0–9, where research with a total score of ≥7 was considered to have good quality [23].

### 2.3. Data Synthesis and Meta-Analysis

The statistical analysis was conducted with STATA (Software for Statistics and Data Science, StataCorp, College Station, TX, USA) software version 17.0 and Review Manager (Nordic Cochrane Centre, Cochrane Collaboration) software version 5.4. All statistical tests were two-sided, and the significance level was defined as *p* < 0.05. We used odds ratios (ORs) as the effect measure with 95% confidence intervals (CIs) for dichotomous data and standardized mean differences (SMDs) with a 95% CI for continuous data. In this case, the continuous outcome was reported in a study as median, range, and interquartile range. We estimated the means and standard deviations using the formula described by Hozo et al. [24]. Heterogeneity was statistically assessed using the Q test and I^2^ statistics. A random synthesis analysis was performed if I^2^ ≥ 50% or the *p* value of the Q tests was less than 0.05. Otherwise, a fixed pooled meta-analysis was performed [25]. We utilized Egger’s test and funnel plots to check for possible biases and funnel plot tests for asymmetry to assess potential publication biases if more than ten trials were included in a single meta-analysis. A sensitivity analysis using leave-one-out cross-validation was performed to test the robustness of the findings.

## 3. Results

**Figure 1:**
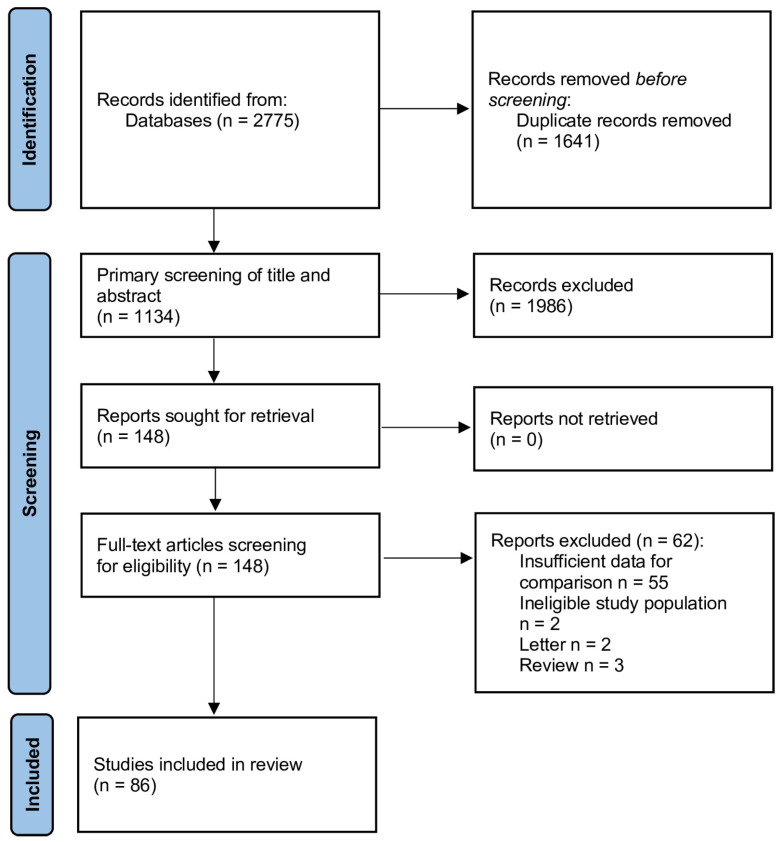
Flow diagram of the search strategy and study selection.

Based on the information depicted in Figure 1, the total count of publications resulting from the database searches amounted to 2775. Out of the total, a total of 1641 duplicate publications were removed. Following an initial screening of titles and abstracts, a total of 148 publications were chosen for a comprehensive evaluation of their complete texts. Subsequently, 62 papers were removed from the analysis since they lacked a control group or did not provide any relevant data. 

**Figure 2:**
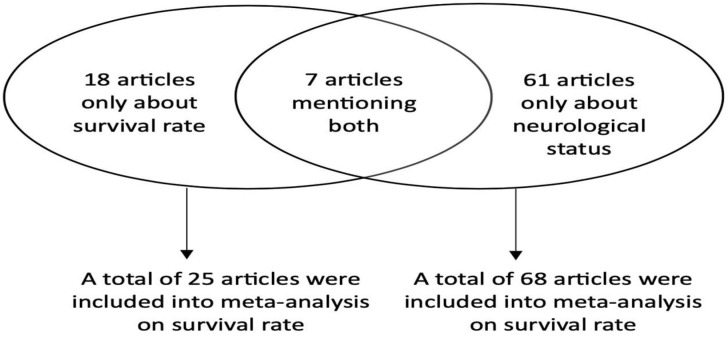
The number and distribution of articles used in the meta-analysis on survival rate and neurological status.

A thorough investigation was undertaken, encompassing a total of 86 studies that met all the stated criteria for inclusion [12,15,26,27,28,29,30,31,32,33,34,35,36,37,38,39,40,41,42,43,44,45,46,47,48,49,50,51,52,53,54,55,56,57,58,59,60,61,62,63,64,65,66,67,68,69,70,71,72,73,74,75,76,77,78,79,80,81,82,83,84,85,86,87,88,89,90,91,92,93,94,95,96,97,98,99,100,101,102,103,104,105,106,107,108]. The aforementioned papers were then incorporated into the meta-analysis. Among those articles, 7 provided data on both the survival rate and neurological outcome, while the other 18 and 61 articles only mentioned information on the survival rate and neurological status, respectively (Figure 2).

Their overall quality was good, with fourteen studies scoring 9/9 on the NOS, the remaining seventy studies scoring 8/9 and twelve studies scoring 7/9 (Table S2).

### 3.1. Study Populations

Eighty-six articles with 10,845 participants were included, and Table S2 displays the main characteristics of these studies. The majority of studies were prospective studies (*n* = 55), with sample sizes ranging from *n* = 15 to *n* = 793. Overall, 21 studies took place in the Republic of Korea, 15 in Germany, 6 in France, 6 in Austria, 4 in Sweden, 4 in Switzerland, 3 in Japan, 3 in the UK, 2 in China, 2 in Luxemburg, 2 in Portugal, 2 in Spain, 2 in the USA, and 1 each, respectively, in Belgium, the Czech Republic, Israel, Norway, Poland, and Romania. In addition, one study [24] was a multicenter study involving research centers from Denmark, Italy, Poland, the UK, and Spain. 

### 3.2. Meta-Analysis of NSE as a Survival Prognostic Marker

**Figure 3:**
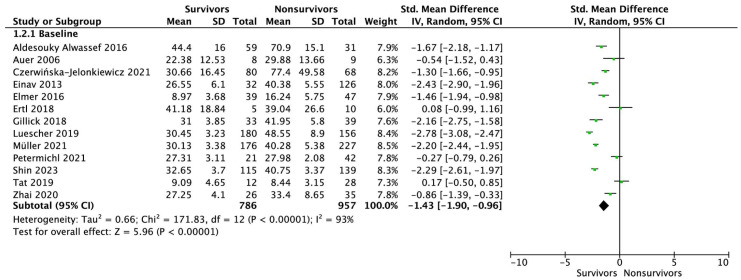
Forest plot of NSE levels among surviving vs. non-surviving patients measured at baseline. The center of each square represents the standardized mean differences for individual trials, and the corresponding horizontal line stands for a 95% confidence interval. The diamonds represent pooled results.

Thirteen studies reported NSE levels immediately after admitting a patient after cardiac arrest. The pooled analysis showed that patients who survived to hospital discharge had statistically significantly lower NSE levels compared with those who died in the hospital (SMD = −1.43; 95% CI: −1.90 to −0.96; *p* < 0.001; Figure 3). The subgroup analysis showed that for OHCAs, NSE levels were statistically significantly lower in survivors compared to non-survivors (SMD = 1.32; 95% CI: −2.02 to −0.63; *p* < 0.001). In contrast, no such relationship was noted for patients with IHCAs (SMD = 0.08; 95% CI: −0.99 to 1.16; *p* = 0.88). The results from the sensitivity analysis did not alter the direction. The funnel plot and Egger’s linear regression test failed to detect a publication bias (Figure S1).

**Figure 4:**
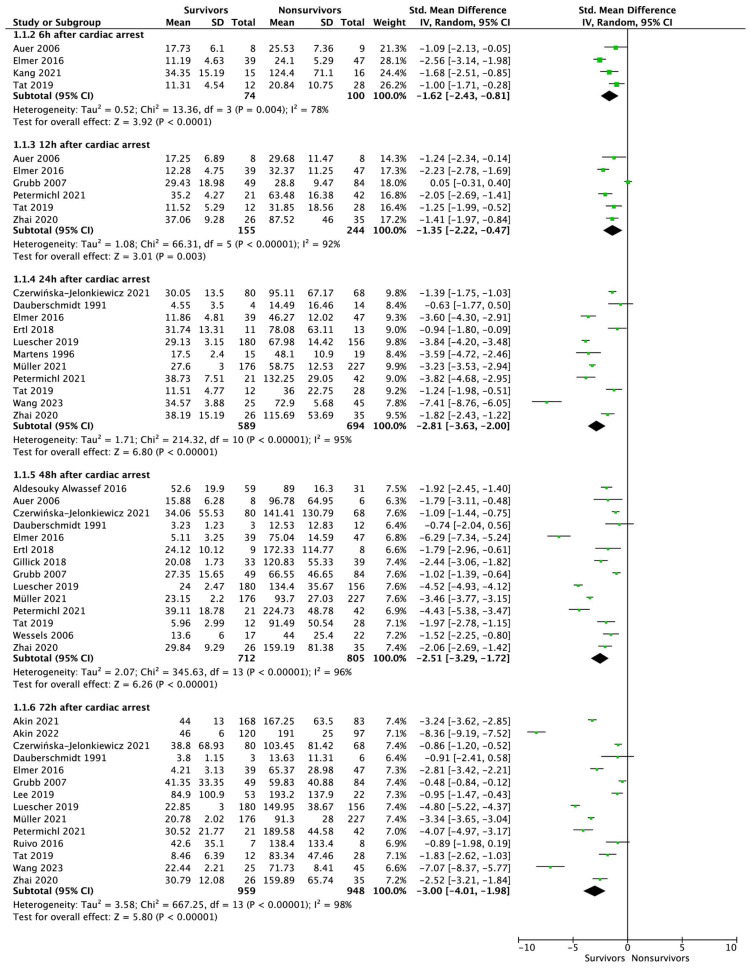
Forest plot of NSE levels among surviving vs. non-surviving patients measured at 6, 12, 24, 48, and 72 h after cardiac arrest. The center of each square represents the standardized mean differences for individual trials, and the corresponding horizontal line stands for a 95% confidence interval. The diamonds represent pooled results.

**Figure 5:**
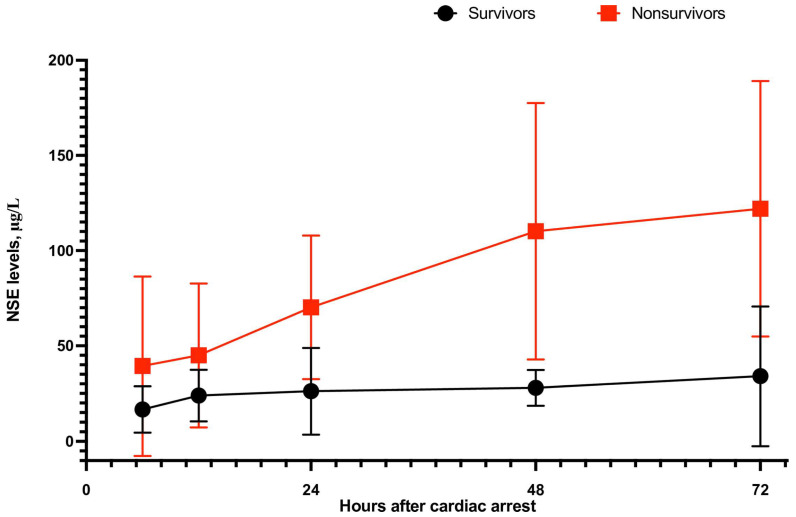
Neuron-specific enolase levels at 6, 12, 24, 48, and 72 h after cardiopulmonary resuscitation in survivors and non-survivors.

Four studies reported NSE levels 6 h after cardiac arrest follow-up. Based on a pooled analysis, the NSE levels of patients who lived and those who died were 16.61 ± 12.13 and 39.36 ± 46.99 μg/L, respectively (Figure 4 and Figure 5; SMD = −1.62; 95% CI: −2.43 to −0.81; *p* < 0.001). Among this group, two articles referred to OHCA patients, and NSE levels were 24.1 ± 16.4 vs. 58.5 ± 66.1 (SMD = −1.30; 95% CI: −1.97 to −0.63; *p* < 0.001).

**Figure 6:**
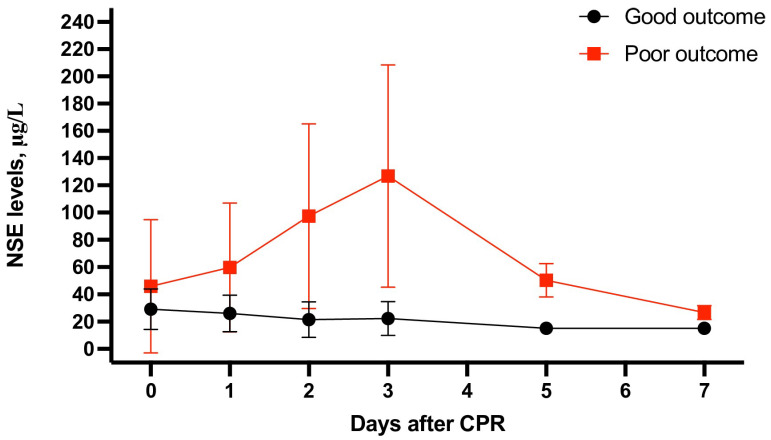
Neuron-specific enolase levels at 0, 1, 2, 3, 5, and 7 days after cardiopulmonary resuscitation in good and poor neurological outcome groups.

Statistically significantly lower levels of NSE were observed for patients who survived compared to patients who did not survive hospital discharge in all other time periods analyzed (Figure 6): 23.9 ± 13.46 vs. 44.99 ± 28.14 μg/L for measurements 12 h after cardiac arrest (SMD = −2.20; 95% CI: −3.51 to −0.88; *p* = 0.001). In a subgroup of patients with OHCAs, NSE values varied among patients who survived vs. those that decreased: 30.4 ± 15.6 vs. 47.8 ± 32.8, respectively (SMD = −1.14; 95% CI: −2.18 to −0.11; *p* = 0.03).

When measured 24 h after cardiac arrest, the NSE values were, respectively, 26.21 ± 22.67 for survivors and 70.22 ± 37.75 μg/L for patients who did not survive hospital discharge (SMD = −2.90; 95% CI: −3.68 to −2.12; *p* < 0.001; Figure 5). Sub-analyses showed that lower NSE values were observed in the group of patients who survived than those in the group of patients who died. This was true for both OHCAs (30.1 ± 9.9 vs. 84.1 ± 47.1; SMD = −2.42; −3.65 to −1.18; *p* < 0.001) as well as IHCAs (31.74 ± 13.31 vs. 78.07 ± 63.11; SMD = −0.94; 95% CI: −1.80 to −0.09; *p* = 0.03).

In the case of measurements 48 h after CA, a pooled analysis showed that NSE levels were 27.97 ± 9.44 vs. 110.21 ± 67.41 μg/L, respectively (SMD = −2.58; 95% CI: −3.34 to −1.82; *p* < 0.001). A similar relationship was observed in the OHCA subgroup (26.7 ± 26.5 vs. 129.5 ± 79.3; SMD = −2.49; 95% CI: −3.67 to −1.30; *p* < 0.001) as well as in the IHCA group 24.12 ± 10.12 vs. 172.33 ± 114.77; SMD = −1.79; 95% CI: −2.96 to −0.61; *p* = 0.003).

When NSE was measured 72 h after cardiac arrest, we also observed statistically significant disparities in NSE values between survivors and decompensated patients, both in terms of all the studies analyzed (34.08 ± 36.56 vs. 122.03 ± 67.15 μg/L; SMD = −2.98; 95% CI: −3.95 to −2.01; *p* < 0.001) and in the sub-analysis of OHCA patients (35.9 ± 37.5 vs. 129.1 ± 66.7; SMD = −3.03; 95% CI: −4.22 to −1.83; *p* < 0.001).

### 3.3. Meta-Analysis of NSE as a Neurological Prognostic Marker

The pooled analysis of NSE levels measured at baseline (after ROSC) among patients who survived with good vs. poor neurological outcomes varied and amounted to 28.89 ± 14.54 vs. 45.96 ± 48.01 μg/L, respectively (SMD = −1.26; 95% CI: 1–59 to −0.93; *p* < 0.001; Figure 6 and Figure S2). The subgroup analysis among OHCA patients showed that NSE levels were 28.68 ± 15.46 and 47.90 ± 38.19 μg/L, respectively (SMD = −1.36; 95% CI: −1.93 to −0.78; *p* < 0.001).

The measurement of NSE in all cohorts on day 1 after ROSC showed statistically significant differences between the groups with good and poor neurological statuses: 25.99 ± 13.34 vs. 59.83 ± 47.24 μg/L (SMD = −1.99; 95% CI: −2.90 to −1.60; *p* < 0.001). A similar relationship was also observed in the subgroup of patients with OHCAs (29.33 ± 14.52 vs. 73.05 ± 58.66 μg/L; SMD = −2.25; 95% CI: −2.90 to −1.60; *p* < 0.001; Figure S3).

The measurement of NSE on day 2 among the entire cohort of patients as well as in the group of OHCA patients varied between patients who survived cardiac arrest with good and poor neurological outcomes: 21.45 ± 13.05 vs. 97.29 ± 67.79 μg/L (SMD = −2.88; 95% CI: −3.30 to −2.46; *p* < 0.001; Figure 5) and 23.09 ± 14.45 vs. 112.01 ± 70.53 μg/L (SMD = −3.39; 95% CI: −4.08 to −2.71; *p* < 0.001), respectively (Figure S4).

The measurement of NSE on day 3 after cardiac arrest showed statistically significant differences among the study groups both in the entire cohort (22.26 ± 12.43 vs. 126.83 ± 81.63; *p* < 0.001) and in the sub-analysis for OHCA patients (24.82 ± 14.07 vs. 139.34 ± 88.02; *p* < 0.001; Figure S5).

A similar relationship was observed in the aspect of measurements on days 5 and 7 after cardiac arrest (Table 1).

The peak NSE levels in good and poor neurological outcome groups were reported among eight studies. The pooled analysis showed that lower levels of NSE were observed in the good neurological outcome group (27.16 ± 11.23 μg/L) than in the poor neurological outcome group (111.66 ± 91.79 μg/L; *p* < 0.001). The sub-analysis showed similar dependencies among OHCA (29.26 ± 12.8 vs. 119.53 ± 90.34; *p* = 0.02) and IHCA patients (24.78 ± 4.09 vs. 105.77 ± 110.66; *p* = 0.008; Figure S6).

## 4. Discussion

The role of NSE was highlighted in the European Resuscitation Council and European Society of Intensive Care Medicine guidelines for post-resuscitation care published in 2021, in which an NSE concentration above 60 µg/L was one of the factors that predicted the likelihood of a poor prognosis. Moreover, the mentioned guideline indicated that increased NSE concentrations between 24 and 48 h (or 72 h), combined with a high isolated NSE value at 48 and 72 h, were associated with a poor prognosis [109]. NSE concentration can also be used to assess whether signs of hypoxic ischemic encephalopathy can be observed with head computed tomography [110]. Taking into account the above, the aim of this meta-analysis, i.e., demonstrating the usefulness of NSE testing in predicting survival in patients after a cardiac arrest, was even more clinically important. The results of the meta-analysis indicated that the high difference in NSE values between survivors and non-survivors may be an additional argument for popularizing the use of biomarkers in patients after cardiac arrests. Rapid patient stratification and the identification of a cohort of patients with a good prognosis may allow for the optimization of early care in ICU units. Biomarkers could also show us patients for whom rehabilitation would bring the greatest clinical improvement [111]. The question about the usefulness of NSE is still valid, especially in the context of the advantage of NSE over other biomarkers. This topic is still open, as suggested by the results of one of the meta-analyses covering a total of 86 studies with 10,567 patients, which indicated that NfL, followed by tau, has greater diagnostic accuracy in predicting favorable vs. unfavorable neurologic outcomes compared with NSE, S100β, glial fibrillary acidic protein (GFAP), and ubiquitin-C-terminal-hydrolase-L1 (UCH-L1) [112].

### 4.1. Searching for New Biomarkers and Regulatory Approaches

The search for new biomarkers indicating significant brain damage during the course of hypoxia, e.g., as a result of sudden cardiac arrest, is the subject of intensive research. New biomarkers with high specificity and sensitivity are being searched for. In a recently published study, Fink et al. analyzed the predictive properties of biomarkers (glial fibrillary acidic protein (GFAP), ubiquitin carboxyl-terminal esterase L1 (UCH-L1), neurofilament light (NfL), and tau concentrations) in pediatric patients after cardiac arrest. Measurements were made between 1 and 3 days after cardiac arrest. In the cited cohort study, NfL was a biomarker that was particularly useful in predicting an unfavorable prognosis (death and significant functional impairment) one year after cardiac arrest [113,114,115]. NfL was also the subject of a recently published meta-analysis that demonstrated its particular utility in predicting neurological statuses when measured 72 h after cardiac arrest. Based on the results obtained from 804 patients, the sensitivity and specificity of NfL after 72 h were determined to be 90% and 98%, respectively [116]. The usefulness of this biomarker was also demonstrated in another meta-analysis among patients with concussions [117]. A promising direction is also the search for biomarkers of non-protein origins, e.g., micro-RNA (miRNA) from extracellular vesicles (EVs). Shen et al. showed, among other things, that miR-124 determined 6 h after resuscitation correlated with the patient’s clinical condition at the time of discharge [118].

It is worth mentioning the decision of the American regulatory body, the Food and Drug Administration, which, in 2018, approved the use of Brain Trauma Indicator (BTI) and UCH-L1 and GFAP in the process of determining the need to perform computed tomography of the head after a mild traumatic brain injury [119]. Despite the dissemination of knowledge about their predictive abilities, in the opinion of physicians, their measurement was still not common. Physicians are also skeptical about the possibility of basing the prognosis only on biomarker concentration values, and it is also necessary to perform electrophysiological tests and neuroimaging [120]. The need for an interdisciplinary approach in neuroprognostication has also also indicated in the Canadian Cardiovascular Society Position Statement, in which biomarkers are just one of many indicated aspects that should be taken into account when determining patient prognoses [121,122]. Other sources also point to the need for an interdisciplinary approach [123].

### 4.2. Obstacles to Implementing NSE into Routine Clinical Practice

Another issue that may limit the widespread use of biomarkers in prognosis estimation is the determination of the cut-off level (significant vs. non-significant) [124]. To avoid a self-fulfilling prophecy, any prediction of patient prognoses should be based on a multi-criterion approach, taking into account clinical assessments by an interdisciplinary team and using neuroimaging and EEG techniques. The adaptation of guidelines is one of the basic steps that can lead to the popularization of biomarkers. Additionally, laboratories should expand the scope of testing when a new predictive biomarker is introduced based on the guidelines. Biomarker determination should also be adequately reimbursed from public funds or by private insurers (depending on the specific characteristics of the healthcare system). This meta-analysis was based on observational studies, mostly prospective and, to a lesser extent, retrospective. Therefore, it is important to remember all the limitations of observational studies. Another issue may have been differences in the biomarker measurement technique. Various sites were included in the studies, and central analyses were generally not used, which may have limited the comparability of the results. Nevertheless, the purpose of using biomarkers, including NSE, in predicting outcomes is to allow for quick and effective modifications in the therapeutic process.

### 4.3. NSE and Prediction of Neurological Status

Moreover, our meta-analysis showed that the higher the NSE value, the worse the patient’s prognosis in terms of neurological status. Of particular importance were the results indicating that high NSE concentrations on the third day after sudden cardiac arrest may be particularly useful in predicting neurological outcomes. However, it is worth remembering that hemolysis may lead to NSE levels that are too high, which are also released from cells other than nerve cells, e.g., red blood cells. Additionally, hemolysis tests are not routinely performed on resuscitated patients. An alternative to NSE in this context may be the previously mentioned neurofilament light chain (NfL), an element of the cytoskeleton of nerve cells. Compared to NSE, NfL has not been identified in cells other than neuronal cells. In this context, Abdi Isse et al. [125] showed that high free hemoglobin at admission was associated with higher NSE concentrations after 48 h, but without affecting the predictive abilities of NSE and NfL. Therefore, the effect of hemolysis described in the literature and mentioned in the introduction may have less clinical significance than expected. The differences in NfL values between patients with good outcomes compared with those of poor outcomes were nevertheless very clear, as indicated by one of the papers published in 2021, where forty-eight hours after OHCA, the median NfL concentration was 19 pg/mL in patients with a good outcome and 2343 pg/mL in those with a poor outcome, *p* < 0.001 [126].

### 4.4. Limitations

This study had some limitations. Initially, the NSE level data demonstrated significant heterogeneity, with substantial overlap observed between the illness groups and control subjects. Hence, it is imperative to exercise caution when applying this biomarker in clinical settings. Furthermore, high levels of NSE indicated neuronal injuries, although they were not specific to any one disease. In addition, the scope of the meta-analysis was restricted to assessing the mortality prognostic effectiveness of the NSE test. The establishment of a conclusive threshold for NSE could not be ascertained through the process of the meta-analysis. Given the existing recommendations, it is recommended that each institution establish its own distinct set of reference values and thresholds relevant to the particular biomarker under consideration. Since the current meta-analysis was about the diagnostic accuracy/prediction of a biomarker, one may argue that the NOS tool used for the risk of bias assessment was not appropriate. Although the NOS tool has been widely used in a way analogous to our meta-analysis, the risk of bias assessment should be considered with due caution. Moreover, to avoid a self-fulfilling prophecy, any prediction of patient prognosis should be based on a multi-criterion approach. 

## 5. Conclusions

Neuron-specific enolase showed an ability to distinguish survivors from non-survivors (mortality) and neurological outcomes among individuals who had experienced cardiac arrest. This study demonstrated the ability to distinguish fatality rates and neurological outcomes, both during the time of admission and at various time intervals following cardiac arrest. The use of NSE in a multimodal neuroprognostication algorithm has promise in improving the accuracy of prognoses for persons who have undergone cardiac arrest, but further studies are warranted. 

## Figures and Tables

**Table 1:** Pooled analysis of Neuron-Specific Enolase (NSE) levels among good and poor neurological outcome groups.

Measurement Period after Cardiac Arrest	No. of Studies	Mean ± SD		Events		Heterogeneity between Trials		*p*-Value for Differences across Groups
		Good Outcome	Poor Outcome	SMD	95% CI	*p*-Value	I^2^ Statistics	
**Neuron-Specific Enolase (NSE) on day 0 (μg/L)**								
All trials	29	28.89 ± 15.46	45.96 ± 48.01	−1.26	−1.59 to −0.93	<0.001	94%	<0.001
OHCA	17	28.68 ± 15.46	47.90 ± 38.19	−1.36	−1.93 to −0.78	<0.001	95%	<0.001
**Neuron-Specific Enolase (NSE) on day 1 (μg/L)**								
All trials	35	25.99 ± 13.34	59.83 ± 47.24	−1.99	−2.36 to −1.62	<0.001	95%	<0.001
OHCA	16	29.33 ± 14.52	73.05 ± 58.66	−2.25	−2.90 to −1.60	<0.001	96%	<0.001
**Neuron-Specific Enolase (NSE) on day 2 (μg/L)**								
All trials	41	21.45 ± 13.05	97.29 ± 67.79	−2.88	−3.30 to −2.46	<0.001	96%	<0.001
OHCA	21	23.09 ± 14.45	112.01 ± 70.53	−3.39	−4.08 to −2.71	<0.001	97%	<0.001
**Neuron-Specific Enolase (NSE) on day 3 (μg/L)**								
All trials	40	22.26 ± 12.43	126.83 ± 81.63	−3.09	−3.52 to −2.45	<0.001	96%	<0.001
OHCA	23	24.82 ± 14.07	139.34 ± 88.02	−3.04	−3.62 to −2.46	<0.001	97%	<0.001
**Neuron-Specific Enolase (NSE) on day 5 (μg/L)**								
All trials	2	15.16 ± 3.43	50.37 ± 12.23	−4.16	−5.01 to −3.32	0.02	81%	<0.001
OHCA	2	15.16 ± 3.43	50.37 ± 12.23	−4.16	−5.01 to −3.32	0.02	81%	<0.001
**Neuron-Specific Enolase (NSE) on day 7 (μg/L)**								
All trials	3	15.17 ± 3.99	26.74 ± 5.08	−3.24	−3.60 to −2.88	0.19	40%	<0.001
OHCA	3	15.17 ± 3.99	26.74 ± 5.08	−3.24	−3.60 to −2.88	0.19	40%	<0.001
**Peak of Neuron-Specific Enolae (μg/L)**								
All trials	7	27.16 ± 11.23	111.66 ± 91.79	−2.14	−3.13 to −1.15	<0.0001	98%	<0.001
OHCA	3	29.26 ± 12.80	119.53 ± 90.34	−2.59	−4.81 to −0.37	<0.001	99%	0.02
IHCA	2	24.78 ± 4.09	105.77 ± 110.66	−1.89	−3.28 to −0.49	<0.001	92%	0.008

Legend: CI: confidence interval; IHCA: in-hospital cardiac arrest; OHCA: out-of-hospital cardiac arrest; SMD: standardized mean difference.

## Data Availability

The data that support the findings of this study are available on request from the corresponding author (L.S.).

## Supplementary Materials

The following supporting information can be downloaded at: https://www.mdpi.com/article/10.3390/jcm12247655/s1, Table S1: Search strategy; Table S2: Baseline characteristics of included trials; Figure S1: Funnel plot analysis. Funnel plot analysis showing asymmetrical funnel plot for NSE as a survival prognostic marker; Figure S2: Forest plot of NSE levels among good vs. poor neurological outcomes measured at baseline; Figure S3: Forest plot of NSE levels among good vs. poor neurological outcomes measured one day after cardiopulmonary resuscitation; Figure S4: Forest plot of NSE levels among good vs. poor neurological outcomes measured two days after cardiopulmonary resuscitation; Figure S5: Forest plot of NSE levels among good vs. poor neurological outcomes measured three days after cardiopulmonary resuscitation; Figure S6: Forest plot of peak NSE levels among good vs. poor neurological outcome groups.
