# Supplementary material for: Predictive Performance of Neuron-Specific Enolase (NSE) for Survival after Resuscitation from Cardiac Arrest: A Systematic Review and Meta-Analysis
Source: J Clin Med. 2023 Dec 13;12(24):7655. doi: 10.3390/jcm12247655 (PMC10744223; doi:10.3390/jcm12247655)
Supplement: Supplementary file 1 [file jcm-12-07655-s001.zip › jcm-2721791-supplementary.pdf]

Predictive performance of neuron-specific enolase (NSE) for survival after resuscitation from cardiac arrest: a systematic review and meta-analysis

Supplementary Digital File

Content:

Table S1. Search Strategy....................2

Table S2: Baseline characteristics of included trials....................3

Figure S1: Funnel-plot analysis. Funnel-plot analysis showing asymmetrical funnel plot for NSE as a survival prognostic marker....................10

Figure S2: Forest plot of NSE levels among good vs. poor neurological outcome measured at baseline....................11

Figure S3: Forest plot of NSE levels among good vs. poor neurological outcome measured one day after cardiopulmonary resuscitation....................12

Figure S4: Forest plot of NSE levels among good vs. poor neurological outcome measured two days after cardiopulmonary resuscitation....................13

Figure S5: Forest plot of NSE levels among good vs. poor neurological outcome measured three days after cardiopulmonary resuscitation....................14

Figure S6: Forest plot of peak of NSE levels among good vs. poor neurological outcome groups.15

Table S1. Search Strategy

**<u>PubMed:</u>**<br>(((((((("Heart Arrest"[Mesh] OR "Death, Sudden"[Mesh]) OR "Cardiac arrest"[TIAB]) OR "heart arrest"[TIAB]) OR "cardiopulmonary arrest"[TIAB]) OR "sudden cardiac death"[TIAB]) OR "sudden cardiac deaths"[TIAB]) OR "sudden death"[TIAB]) OR "sudden deaths"[TIAB]) AND (((((("Cardiopulmonary Resuscitation"[Mesh] OR "Heart Massage"[Mesh]) OR CPR[TIAB]) OR "cardiopulmonary resuscitation"[TIAB]) OR "chest compression"[TIAB]) OR "chest compressions"[TIAB]) OR (resuscitate[TIAB] OR resuscitated[TIAB] OR resuscitates[TIAB] OR resuscitating[TIAB] OR resuscitation[TIAB] OR resuscitational [TIAB] OR resuscitationists[TIAB] OR resuscitationperiod[TIAB] OR resuscitations[TIAB] OR resuscitative[TIAB] OR resuscitator[TIAB] OR resuscitator's[TIAB] OR resuscitators[TIAB] OR resuscitators'[TIAB] OR resuscitatory[TIAB]))) AND (("neuron-specific enolase"[Mesh] OR neuron specific enolase [TIAB] OR NSE[TIAB]) NOT ("animals"[MeSH Terms]) NOT ("letter"[pt] OR "comment"[pt] OR "editorial"[pt]))
**<u>EMBASE:</u>**<br>'heart arrest'/exp OR 'sudden death'/exp OR 'cardiac arrest':ab,ti OR 'heart arrest':ab,ti OR 'cardiopulmonary arrest':ab,ti OR 'sudden cardiac death':ab,ti OR 'sudden cardiac deaths':ab,ti OR 'sudden death':ab,ti OR 'sudden deaths':ab,ti AND ('resuscitation'/exp OR 'heart massage'/exp OR cpr:ab,ti OR 'cardiopulmonary resuscitation':ab,ti OR 'chest compression':ab,ti OR 'chest compressions':ab,ti OR resuscitat* OR 'heart massage':ab,ti OR 'cardiac massage':ab,ti) AND (‘neuron-specific enolase'/exp OR neuron specific enolase:ab,ti OR NSE:ab,ti NOT ('animal'/exp) NOT ([editorial]/lim OR [letter]/lim) AND [embase]/lim
**<u>COCHRANE:</u>**<br>([mh "Heart Arrest"] or [mh "Death, Sudden"] or "Cardiac arrest":ab,ti or "heart arrest":ab,ti or "cardiopulmonary arrest":ab,ti or "sudden cardiac death":ab,ti or "sudden cardiac deaths":ab,ti or "sudden death":ab,ti or "sudden deaths":ab,ti) and ([mh "Cardiopulmonary Resuscitation"] or [mh "Heart Massage"] or CPR:ab,ti or "cardiopulmonary resuscitation":ab,ti or "chest compression":ab,ti or "chest compressions":ab,ti or resuscitat* or "heart massage":ab,ti or "cardiac massage":ab,ti) and ([mh "neuron-specific enolase"/IS] or neuron specific enolase:ab,ti or NSE:ab,ti
**<u>WEB of SCIENCE</u>**<br># 3 #2 AND #1<br># 2 TS=(neuron-specific enolase or neuron specific enolase or NSE*)<br># 1 TS=(cardiac arrest or sudden death or cardiopulmonary arrest)

Table S2: Baseline characteristics of included trials.

Study	Country	Study design	CA type	Study group	No. of patients	Age	Sex, male	ROSC, min	Shockable primary rhythm	Witnessed arrest (%)	NOS score
Adler et al., 2022	Germany	RS	OHCA	GNO	30	61 ± 11	24 (80.0%)	15.5 ± 10.9	28 (93.3%)	22 (73.3%)	8
				PNO	23	67 ± 13	20 (86.9%)	26.4 ± 20.1	17 (73.9%)	8 (34.8%)	
Ahn et al., 2022	Republic of Korea	RS	OHCA	GNO	46	55.3 ± 7.4	36 (78.3%)	NS	23 (50.0%)	37 (80.4%)	9
				PNO	60	56.9 ± 7.6	41 (68.3%)	NS	4 (6.7%)	34 (56.7%)	
Akin et al., 2021	Germany	PS	OHCA	Survivors	168	59 ± 13	134 (79.8%)	22±16	132 (78.6%)	149 (88.7%)	9
				Non-survivors	83	68 ± 12	34 (40.9%)	24±16	54 (65.1%)	65 (78.3%)	
				GNO	102	57 ± 13	81 (79.4%)	21±16	81 (79.4%)	92 (90.2%)	
				PNO	149	66 ± 13	116 (77.9%)	23±16	105 (70.5%)	122 (81.9%)	
Akin et al., 2022	Germany	PS	OHCA	Survivors	120	58 ± 16	98 (81.7%)	15 (15-25)	84 (70.0%)	101 (84.2%)	9
				Non-survivors	97	67 ± 12	69 (71.1%)	25 (15-35)	31 (31.9%)	73 (75.3%)	
				GNO	59	56 ± 14	46 (77.9%)	15 (14-25)	49 (83.1%)	50 (84.7%)	
				PNO	158	65 ± 14	121 (76.6%)	20 (15-30)	66 (41.8%)	124 (78.5%)	
Aldesouky Alwassef et al., 2016	Egypt	PS	IHCA / OHCA	Survivors	59	NS	NS	NS	NS	NS	8
				Non-survivors	31	NS	NS	NS	NS	NS	
				GNO	41	NS	NS	NS	NS	NS	
				PNO	49	NS	NS	NS	NS	NS	
Andersson et al., 2022	Sweden	RS	IHCA	GNO	98	71 (63-77)	58 (59.2%)	7 (5-15)	29 (29.6%)	90 (91.8%)	9
				PNO	146	75 (67-79)	92 (63.0%)	11 (6-20)	21 (14.3%)	118 (80.8%)	
			OHCA	GNO	175	65 (53-72)	138 (78.9%)	15 (10-25)	135 (77.1%)	149 (85.1%)	
				PNO	374	70 (62-77)	264 (70.6%)	30 (20-45)	128 (34.2%)	268 (71.7%)	
Auer et al., 2006	Austria	RS	IHCA / OHCA	Survivors	8	NS	NS	NS	NS	NS	7
				Non-survivors	9	NS	NS	NS	NS	NS	
Barbella et al., 2020	Switzerland	RS	IHCA	GNO	10	65.5 ± 17.8	7 (70.0%)	23.0 ± 11.6	7 (70.0%)	NS	8
				PNO	97	63.6 ± 17.0	65 (67.0%)	25.3 ± 12.8	40 (41.2%)	NS	
Benghanem et al., 2022	France	PS	OHCA	GNO	18	62 (54-71)	10 (55.6%)	NS	12 (66.7%)	NS	8
				PNO	64	66.5 (51-76)	41 (64.1%)	NS	16 (25.4%)	NS	
Choi et al., 2016	Republic of Korea	PS	OHCA	GNO	46	50.6 ± 16.0	37 (80.4%)	22.3 ± 12.2	33 (71.7%)	38 (82.6%)	9
				PNO	73	55.8 ± 15.2	44 (60.3%)	36.9 ± 17.9	15 (20.5%)	53 (72.6%)	
Chong et al., 2020	Republic of Korea	PS	OHCA	GNO	20	51.9 ± 16.95	19 (42.2%)	NS	10 (22.2%)	16 (35.6%)	8
				PNO	25	55.0 ± 18.58	18 (40.0%)	NS	0 (0.0%)	13 (28.9%)	
	USA	PS		GNO	42	58 ± 18	24 (57.1%)	20 (10-30)	21 (50.0%)	NS	8

Chung-Esaki et al., 2018			IHCA / OHCA	PNO	55	61 ± 17	35 (63.6%)	20 (15-25)	15 (27.3%)	NS	
Czerwińska-Jelonkiewicz et al., 2021	Multicountry	PS	OHCA	Survivors	80	59.5	21 (27.3%)	16.6 ± 2.4	65 (86.7%)	NS	8
				Non-survivors	68	65.57 ± 14.87	20 (29.4%)	24.2 ± 12.6	37 (54.4%)	NS	
Dauberschmidt et al., 1991	Germany	PS	IHCA / OHCA	Survivors	4	NS	NS	NS	NS	NS	7
				Non-survivors	14	NS	NS	NS	NS	NS	
Daubin et al., 2011	France	PS	IHCA / OHCA	GNO	25	50 ± 17	19 (76.0%)	22.5 ± 15.7	14 (56.0%)	20 (80.0%)	8
				PNO	72	60 ± 15	56 (77.8%)	25.7 ± 26.6	21 (29.2%)	57 (79.2%)	
Deye et al., 2020	France	PS	IHCA / OHCA	GNO	109	55 (45-67)	84 (77.1%)	NS	80 (73.4%)	103 (94.5%)	9
				PNO	221	63 (52-76)	146 (66.1%)	NS	45 (20.4%)	209 (94.6%)	
Einav et al., 2013	Israel	PS	OHCA	Survivors	32	65.8 ± 14.2	26 (81.3%)	NS	19 (59.4%)	NS	8
				Non-survivors	126	76.4 ± 15.2	78 (61.9%)	NS	15 (11.9%)	NS	
Elmer et al., 2016	USA	PS	IHCA / OHCA	Survivors	39	56 (43-56)	21 (53.8%)	NS	24 (61.5%)	NS	8
				Non-survivors	47	56 (45-56)	29 (61.7%)	NS	15 (31.9%)	NS	
Ertl et al., 2018	Germany	PS	IHCA	Survivors	23	59	NS	17 (73.9%)	NS	NS	8
				Non-survivors	26	69	NS	12 (52.2%)	NS	NS	
Gillick et al., 2018	UK	RS	OHCA	Survivors	33	NS	NS	NS	NS	NS	7
				Non-survivors	39	NS	NS	NS	NS	NS	
Grubb et al., 2007	UK	PS	OHCA	Survivors	56	NS	NS	NS	NS	NS	7
				Non-survivors	49	NS	NS	NS	NS	NS	
Haertel et al., 2023	Germany	RS	IHCA / OHCA	GNO	25	61.2 ± 20.2	16 (64.0%)	31.9 ± 24.4	15 (60.0%)	NS	8
				PNO	165	60.0 ± 15.1	125 (75.8%)	55.2 ± 38.6	77 (46.7%)	NS	
Hasper et al., 2009	Germany	RS	IHCA / OHCA	GNO	69	60 (52-69)	58 (84.1%)	NS	63 (91.3%)	NS	8
				PNO	102	65 (54-74)	76 (74.5%)	NS	48 (47.1%)	NS	
Hasslacher et al., 2014	Austria	PS	IHCA / OHCA	GNO	65	59 (50-70)	46 (70.8%)	NS	50 (76.9%)	NS	8
				PNO	69	67 (58-76)	53 (76.8%)	NS	29 (42.0%)	NS	
Helwig et al., 2017	Germany	PS	IHCA / OHCA	GNO	39	60.9 ± 14.1	27 (69.2%)	NS	28 (71.8%)	NS	8
				PNO	61	73.0 ± 12.8	35 (57.4%)	NS	21 (34.4%)	NS	
Hermann et al., 2023	France	RS	IHCA / OHCA	GNO	47	58 (45-66)	31 (66.7%)	NS	22 (46.8%)	31 (65.9%)	8
				PNO	134	63 (51-73)	85 (63.4%)	NS	35 (26.1%)	89 (66.4%)	
Jakkula et al., 2019	Finland	PS	OHCA	GNO	78	58 ± 13	65 (83.3%)	17 (14-22)	NS	NS	8
				PNO	40	63 ± 13	31 (77.5%)	25 (21-31)	NS	NS	
Kaneko et al., 2017	Japan	PS	OHCA	GNO	20	62 (52-66)	17 (85.0%)	6 (5-10)	17 (85.0%)	16 (80.0%)	8
				PNO	23	71 (57-79)	16 (69.6%)	7 (0-15)	11 (47.8%)	16 (69.6%)	

Kang et al., 2021	Republic of Korea	PS	OHCA	GNO	36	53.3 ± 19.0	28 (77.8%)	NS	20 (55.6%)	27 (75.0%)	8
				PNO	49	53.3 ± 18.1	34 (69.4%)	NS	4 (8.1%)	26 (53.1%)	
Kang et al., 2021b	Republic of Korea	PS	OHCA	Survivors	15	53 (28-77)	13 (86.7%)	NS	8 (53.3%)	11 (73.3%)	8
				Non-survivors	16	54 (16-78)	7 (43.8%)	NS	1 (6.3%)	5 (31.3%)	
Kim et al., 2012	Republic of Korea	RS	OHCA	GNO	11	NS	NS	NS	NS	NS	7
				PNO	32	NS	NS	NS	NS	NS	
Kim et al., 2020	Republic of Korea	PS	IHCA / OHCA	GNO	19	58.3 ± 19.7	10 (52.6%)	13.4 ± 11.3	7 (36.8%)	NS	8
				PNO	64	61.5 ± 18.6	40 (62.5%)	23.2 ± 15.8	7 (10.9%)	NS	
Kim et al., 2020b	Republic of Korea	RS	OHCA	GNO	34	52.3 ± 5.4	25 (73.5%)	20.2 ± 4.8	22 (64.7%)	27 (79.4%)	9
				PNO	75	53.0 ± 4.3	49 (65.3%)	36.3 ± 3.5	15 (20.0%)	38 (50.7%)	
Kim et al., 2023	Republic of Korea	RS	IHCA / OHCA	GNO	9	41.0 ± 12.7	5 (55.6%)	NS	4 (57.1%)	9 (100.0%)	8
				PNO	16	51.2 ± 13.8	13 (81.2%)	NS	5 (35.7%)	13 (81.2%)	
Kirsch et al., 2021	Germany	RS	IHCA / OHCA	GNO	22	61.5 ± 3.9	15 (68.2%)	19.0 ± 4.5	14 (63.6%)	NS	8
				PNO	69	64.9 ± 1.7	53 (76.8%)	28.3 ± 2.3	18 (26.1%)	NS	
Kwon et al., 2021	Republic of Korea	PS	OHCA	GNO	13	62.2 ± 17.9	12 (92.3%)	16.5 ± 13.5	8 (61.5%)	11 (84.6%)	9
				PNO	44	71.0 ± 10.8	35 (79.5%)	32.7 ± 17.5	4 (9.1%)	35 (79.5%)	
Maher et al., 2023	UK	RS	OHCA	GNO	23	61	15 (65.2%)	NS	NS	NS	8
				PNO	36	65	27 (75.0%)	NS	NS	NS	
Larsson et al., 2014	Sweden	PS	IHCA / OHCA	GNO	57	63 ± 14	38 (66.7%)	20 ± 14	35 (61.4%)	52 (91.2%)	9
				PNO	68	69 ± 12	45 (66.2%)	24 ± 15	22 (32.4%)	56 (82.3%)	
Lascarrou et al., 2021	France	PS	IHCA / OHCA	GNO	13	61.9 ± 12.7	9 (69.2%)	NS	0 (0.0%)	13 (100.0%)	9
				PNO	88	66.5 ± 14.4	53 (60.2%)	NS	0 (0.0%)	77 (88.5%)	
Leao et al., 2015	Portugal	PS	IHCA / OHCA	GNO	12	59.5 ± 13.3	NS	21.5 ± 18.2	8 (66.7%)	NS	8
				PNO	55	63.0 ± 13.6	NS	26.3 ± 15.8	18 (32.7%)	NS	
Lee et al., 2019	Republic of Korea	PS	OHCA	Survivors	53	57.9 ± 16.9	40 (75.5%)	23.3 ± 17.8	26 (49.1%)	42 (79.3%)	8
				Non-survivors	22	60.8 ± 17.8	13 (59.1%)	30.4 ± 14.6	5 (22.7%)	14 (63.6%)	
				GNO	30	55.9 ± 18.0	22 (73.3%)	19.1 ± 14.8	18 (60.0%)	25 (83.#%)	8
				PNO	45	60.7 ± 16.5	31 (68.9%)	29.6 ± 17.5	13 (28.9%)	31 (68.9%)	
Lee et al., 2021	South Korea	RS	OHCA	GNO	171	54 (44-62)	132 (77.2%)	19 (13-30)	114 (66.7%)	141 (82.5%)	9
				PNO	304	61 (51-71)	214 (70.4%)	34 (22-45)	50 (16.4%)	191 (62.8%)	
Lee et al., 2023	Republic of Korea	RS	OHCA	GNO	55	59 (45-70)	45 (81.8%)	NS	33 (60.0%)	48 (87.3%)	8
				PNO	70	58 (40-69)	48 (68.6%)	NS	7 (10.0%)	27 (38.6%)	
Luescher et al., 2019	Switzerland	PS	OHCA	Survivors	180	NS	NS	NS	NS	NS	8
				Non-survivors	156	NS	NS	NS	NS	NS	
				GNO	150	61 ± 14.8	124 (82.7%)	NS	114 (76.0%)	139 (92.7%)	

				PNO	186	66 ± 13.8	119 (64.0%)	NS	74 (39.8%)	139 (75.1%)	
Maher et al., 2023	UK	RS	OHCA	GNO	23	61	15 (65.2%)	NS	NS	NS	7
				PNO	36	65	27 (75.0%)	NS	NS	NS	
Martens et al., 1996	Balgium	PS	IHCA / OHCA	Survivors	15	55.6 ± 5	6 (40.0%)	NS	NS	NS	7
				Non-survivors	19	54.7 ± 5.7	5 (26.3%)	NS	NS	NS	
Martinez-Losas et al., 2020	Spain	RS	IHCA / OHCA	GNO	174	59.5 ± 14.7	145 (83.3%)	20.4 ± 3.0	150 (86.2%)	170 (97.7%)	8
				PNO	146	64.1 ± 14.4	112 (76.7%)	27.3 ± 2.5	70 (47.9%)	131 (89.7%)	
Müller et al., 2021	Switzerland	PS	IHCA / OHCA	Survivors	176	59.7 ± 14.4	138 (78.4%)	17.3 ± 15.3	93	NS	8
				Non-survivors	227	67.6 ± 13.8	146 (64.3%)	26.4 ± 16.7	132	NS	
Mörtberg et al., 2021	Sweden	PS	IHCA / OHCA	GNO	15	60 (22-78)	12	17 (5-29)	10	13	9
				PNO	16	68.5 (25-84)	9	22 (5-45)	6	12	
Nakstad et al., 2019	Norway	PS	OHCA	GNO	128	59.7 ± 12.4	111	22.2 (16.0)	107	122	8
				PNO	131	62.6 ± 15.1	103	33.0 (19.3)	69	106	
Ok et al., 2021	Korea	PS	IHCA / OHCA	GNO	20	48 (34-65.58)	12 (60.0%)	13 (10-28.8)	13 (65.0%)	16 (80.0%)	8
				PNO	34	60 (44.8-72)	25 (73.5%)	34.5 (16-46.8)	4 (11.8%)	21 (61.8%)	
Oksanen et al., 2009	Finalnd	PS	IHCA / OHCA	GNO	50	60 (47-68)	37	18 (14-21.5)	NS	NS	8
				PNO	40	67 (60-72)	34	20 (16.8-26)	NS	NS	
Park et al., 2019	Korea	RS	OHCA	GNO	39	53 (40-59)	26 (66.7%)	20 (11-34)	23 (59.0%)	19 (48.7%)	8
				PNO	63	57 (48-68)	46 (73.0%)	32 (23-45)	12 (19.0%)	28 (44.4%)	
Petermichl et al., 2021	Germany	PS	OHCA	Survivors	17	NS	NS	NS	NS	NS	8
				Non-survivors	46	NS	NS	NS	NS	NS	
Pfeifer et al., 2005	Germany	PS	IHCA / OHCA	GNO	70	63 (17-90)	NS	24.9 (5-35)	28	38	8
				PNO	27	63.2 (29-87)	NS	20.7 (5-60)	18	21	
Rafecas et al., 2020	Spain	RS	OHCA	GNO	96	55.4 (47.8-63.4)	82 (85.4%)	23 (15-33)	NS	NS	8
				PNO	69	60.1 (53.4-69.8)	56 (81.1%)	30 (22-38)	NS	NS	
Reisinger et al., 2007	Austria	PS	IHCA / OHCA	GNO	90	NS	NS	NS	NS	NS	8
				PNO	87	NS	NS	NS	NS	NS	
Roger et al., 2015	France	RS	OHCA	GNO	27	60 ± 16	NS	NS	21	22	8
				PNO	53	61 ± 17	NS	NS	21	35	

Rossetti et al., 2012	Switzerland	PS	IHCA / OHCA	GNO	28	NS	NS	NS	NS	NS	7
				PNO	33	NS	NS	NS	NS	NS	
Ruivo et al., 2016	Portugal	RS	IHCA / OHCA	Survivors	7	38.4 ± 10.1	5 (71.4%)	22.9 ± 12.9	6 (85.7%)	NS	8
				Non-survivors	8	55.1 ± 13.2	5 (62.5%)	63.8 ± 40.4	4 (50.0%)	NS	
Rundgren et al., 2009	Sweden	PS	IHCA / OHCA	GNO	56	NS	NS	NS	NS	NS	8
				PNO	46	NS	NS	NS	NS	NS	
Ruttmann et al., 2017	Austria	RS	OHCA	GNO	21	37.3 ± 24.4	16	43 ± 14	12 (57.1%)	13 (61.9%)	8
				PNO	32	46.5 ± 26.6	21	71 ± 12	18 (56.3%)	7 (21.9%)	
Ryczek et al., 2021	Poland	PS	OHCA	GNO	22	60 (45-70)	15 (68.2%)	15 (10-25)	20 (90.9%)	NS	8
				PNO	60	67 (62-76.5)	41 (68.3%)	28 (19-45)	34 (56.7%)	NS	
Ryoo et al., 2020	Korea	RS	OHCA	GNO	62	52.6 ± 16.5	47 (75.8%)	NS	31 (50.0%)	53 (85.5%)	8
				PNO	98	62.8 ± 15.9	61 (62.2%)	NS	18 (18.4%)	68 (69.4%)	
Sallam et al., 2012	Egypt	PS	OHCA	Survivors	43	NS	NS	NS	NS	NS	7
				Non-survivors	37	NS	NS	NS	NS	NS	
				GNO	31	NS	NS	NS	NS	NS	
				PNO	49	NS	NS	NS	NS	NS	
Schefold et al., 2009	Germany	PS	IHCA / OHCA	GNO	44	58 (47-66)	35	NS	34	NS	8
				PNO	28	58 (48-70)	19	NS	15	NS	
Schoerkhuber et al., 1999	Austria	PS	IHCA / OHCA	GNO	28	57 ± 11	15 (53.6%)	NS	NS	NS	7
				PNO	28	61 ± 14	18 (64.3%)	NS	NS	NS	
Shin et al., 2023	Republic of Korea	RS	OHCA	Survivors	115	59 (50-72)	82 (71.3%)	NS	49 (42.6%)	83 (72.2%)	8
				Non-survivors	139	70 (57-79)	82 (59.0%)	NS	22 (15.8%)	88 (63.3%)	
Shinozaki et al., 2009	Japan	PS	IHCA / OHCA	GNO	13	59 ± 12	13 (100%)	NS	9	13 (100%)	8
				PNO	67	67 ± 15	35	NS	8	48	
Son et al., 2020	Korea	RS	OHCA	GNO	25	50.5(43-58.1)	20 (80.0%)	NS	16 (64.0%)	21 (84.0%)	8
				PNO	33	55.3 (48.8-61.7)	20 (75.8%)	NS	3 (9.4%)	15 (45.5%)	
Song et al., 2021	Korea	RS	OHCA	GNO	45	57 (42-68)	37 (82.2%)	NS	24 (53.3%)	36 (80.0%)	9
				PNO	61	57 (40.5-76.8)	41 (67.2%)	NS	5 (8.1%)	33 (53.2%)	
Song et al., 2023	Korea	PS	OHCA	GNO	46	53.3 ± 15.5	39 (84.8%)	243 ± 49.9	27 (58.7%)	32 (69.6%)	8
				PNO	54	58.3 ± 16.2	39 (72.2%)	54.4 ± 59.6	9 (16.7%)	32 (59.3%)	
Stammet et al., 2013	Luxemburg	PS	OHCA	GNO	41	61 (29-82)	34	20 (4-60)	36	NS	8
				PNO	34	69 (38-83)	23	30 (12-76)	13	NS	

Stefanizzi et al., 2022	Luxemburg	PS	OHCA	GNO	286	60 (20-90)	238 (83.2%)	20 (0-160)	260 (90.9%)	262 (91.6%)	8
				PNO	256	68 (35-94)	205 (80.1%)	30 (0-170)	170 (66.4%)	223 (87.1%)	
Storm et al., 2012	Germany	PS	IHCA / OHCA	GNO	11	NS	NS	NS	NS	NS	7
				PNO	24	NS	NS	NS	NS	NS	
Sugita et al., 2017	Japan	PS	OHCA	GNO	25	60 (48–71)	24	28.4 ± 22.3	17	18	8
				PNO	103	72 (63–83)	64	46.7 ± 25.0	30	72	
Tat et al., 2019	Romania	PS	OHCA	Survivors	12	58.2 ± 6.2	9 (75.0%)	9.1 ± 4.6	10 (83.3%)	NS	8
				Non-survivors	28	69 (66-76.7)	19 (67.9%)	15 (13-17)	2 (7.1%)	NS	
Vondrakova et al., 2017	Czech Republic	RS	OHCA	GNO	96	NS	NS	NS	NS	NS	8
				PNO	57	NS	NS	NS	NS	NS	
Wang et al., 2023	China	PS	IHCA / OHCA	Survivors	23	58.5 ± 16.1	17 (73.9%)	7 (3-10)	14 (60.9%)	NS	9
				Non-survivors	45	65.0 ± 15.3	28 (62.2%)	15 (8-23)	18 (40.0%)	NS	
Wessels et al., 2006	Germany	PS	IHCA / OHCA	Survivors	17	NS	NS	NS	NS	NS	7
				Non-survivors	22	NS	NS	NS	NS	NS	
Wennervirta et al., 2009	Finland	PS	OHCA	GNO	21	55 (24-74)	16	16 (9-23)	NS	NS	8
				PNO	9	60 (26-77)	8	22 (15-31)	NS	NS	
Wihersaari et al., 2022	Finland	RS	OHCA	Survivors	163	62 (56-70)	139 (85.3%)	17 (11-23)	128 (78.5%)	152 (93.3%)	9
				Non-survivors	86	66 (59-72)	70 (81.4%)	26 (20-31.3)	49 (60.0%)	75 (87.2%)	
				GNO	128	61.5 (55.3-67)	107 (83.6%)	16 (11-23)	106 (82.8%)	123 (96.1%)	
				PNO	121	67 (59-72)	102 (84.3%)	24 (19-31)	71 (58.7%)	104 (86.0%)	
Wolff et al., 2009	Germany	RS	IHCA / OHCA	GNO	28	54 (46-66)	23 (82.1%)	NS	27 (96.4%)	NS	8
				PNO	21	64 (51-70)	16 (76.2%)	NS	14 (66.7%)	NS	
Wurm et al., 2022	Austria	PS	OHCA	GNO	21	56 (43-66)	16	27 (18-33)	19	19	8
				PNO	49	60 (49-70)	37	25.5 (14.75-38.75)	35	37	
Yoon et al., 2023	Korea	RS	OHCA	GNO	57	56 (51-60)	47 (82.5%)	NS	36 (63.2%)	49 (86.0%)	8
				PNO	74	57 (53-61)	50 (67.6%)	NS	9 (2.2%)	31 (41.9%)	
You et al., 2019	Korea	PS	OHCA	GNO	18	47.9 ± 6.8	15 (83.3%)	NS	11 (61.1%)	14 (77.8%)	8
				PNO	16	49.5 ± 5.1	7 (43.8%)	NS	1 (6.3%)	9 (56.3%)	
Zellner et al., 2013	Germany	RS	IHCA / OHCA	GNO	61	61 ± 14	52 (85.2%)	NS	50 (82.0%)	52 (85.2%)	8
				PNO	62	61 ± 15	46 (74.2%)	NS	31 (50.8%)	40 (64.5%)	
	China	PS	OHCA	Survivors	26	53.4 ± 16.3	20 (76.9%)	21.3 ± 5.3	10 (38.5%)	5 (19.2%)	8

Zhai et al., 2020				Non-survivors	35	71.0 ± 16.1	20 (57.1%)	29.6 ± 9.6	9 (25.7%)	4 (11.4%)	

Legend: GNO: good neurological outcome (CPC ≤ 2); NS: not specified; PNO: poor neurological outcome (CPC ≥ 3); PS: prospective study design; RS: retrospective study design.

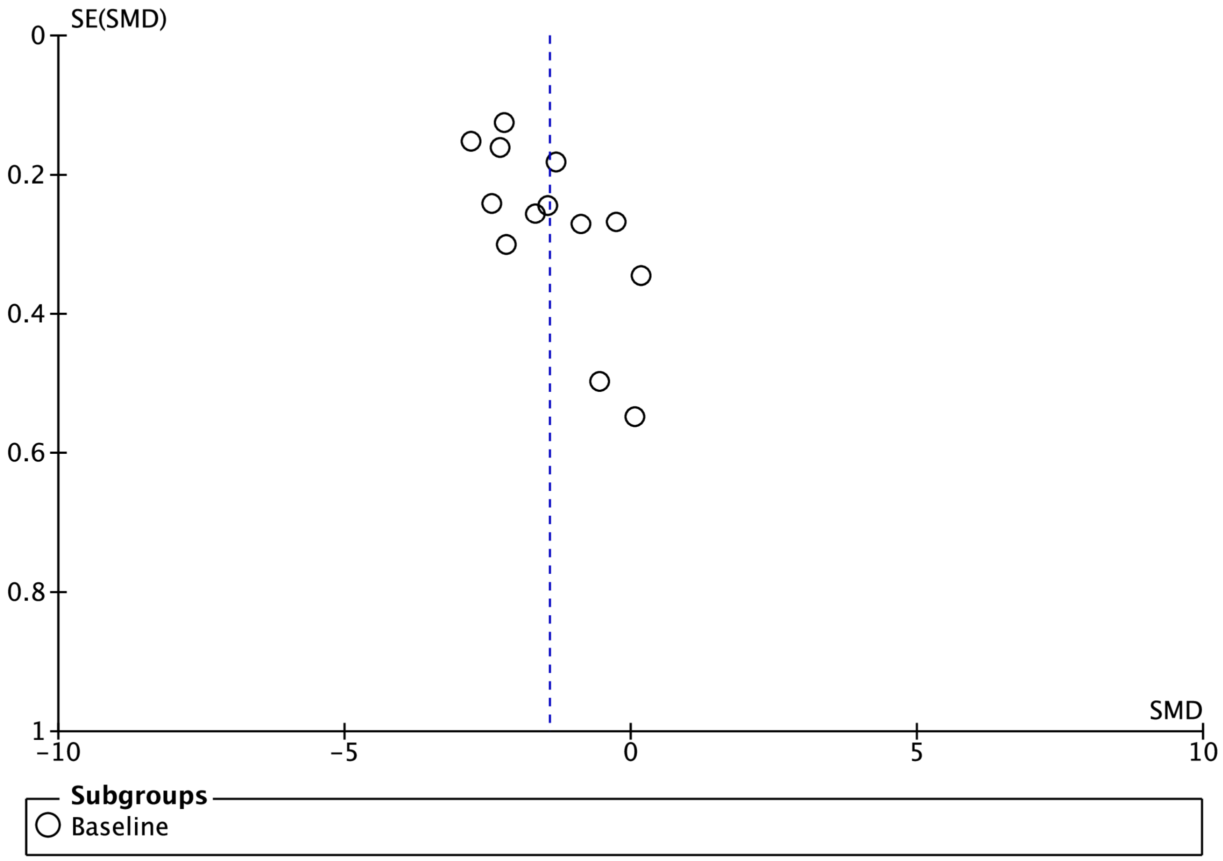

Figure S1: Funnel-plot analysis. Funnel-plot analysis showing asymmetrical funnel plot for NSE as a survival prognostic marker.

Study or Subgroup	Good outcome (CPC ≤ 2) Mean	SD	Total	Poor outcome (CPC ≥ 3) Mean	SD	Total	Weight	Std. Mean Difference IV, Random, 95% CI
**1.1.1 OHCA**								
Adler 2022	79.7	6.9	30	81.8	17.3	23	3.4%	−0.17 [−0.71, 0.38]
Choi 2016	26.49	9.4	38	39.17	36.8	73	3.6%	−0.41 [−0.81, −0.02]
Kang 2021	27.33	11.58	36	101.75	71.1	49	3.5%	−1.35 [−1.83, −0.87]
Kim 2012	29.4	7.5	11	30.8	14	32	3.3%	−0.11 [−0.79, 0.58]
Kwon 2021	53.49	50.73	13	89.34	110.33	44	3.3%	−0.35 [−0.97, 0.27]
Luescher 2019	29.58	3.38	150	46.23	8.05	186	3.7%	−2.59 [−2.89, −2.30]
Nakstad 2019	22.66	3.85	110	29.94	5.97	27	3.5%	−1.67 [−2.13, −1.20]
Park 2019	25.51	3.85	39	23.69	3.3	63	3.6%	0.51 [0.11, 0.92]
Ruttmann 2017	34.7	14.2	21	88.4	42.7	32	3.3%	−1.54 [−2.16, −0.91]
Ryczek 2021	24.63	3.48	22	38.54	5.49	60	3.3%	−2.73 [−3.38, −2.08]
Sallam 2012	29.8	6.7	31	39.54	17.86	49	3.5%	−0.66 [−1.12, −0.20]
Song 2021	24.03	3.03	45	50.88	10.98	61	3.4%	−3.11 [−3.69, −2.54]
Song 2023	33.05	4.95	46	43.18	7.03	54	3.5%	−1.63 [−2.09, −1.18]
Sugita 2017	9.7	6.8	25	32.64	7.82	103	3.4%	−2.99 [−3.56, −2.41]
Wennervirta 2009	19.65	6.1	21	19.15	4.46	9	3.1%	0.09 [−0.70, 0.87]
Yoon 2023	24.03	2.28	57	48.2	11.35	74	3.5%	−2.77 [−3.26, −2.29]
You 2019	26.75	3.6	18	37.35	9.3	16	3.1%	−1.50 [−2.28, −0.73]
**Subtotal (95% CI)**			**713**			**955**	**58.1%**	**−1.36 [−1.93, −0.78]**
Heterogeneity: $Tau^2 = 1.38$; $Chi^2 = 354.18$, df = 16 (P < 0.00001); $I^2 = 95\%$								
Test for overall effect: Z = 4.63 (P < 0.00001)								
**1.1.3 Mix OHCA and IHCA**								
Aldesouky Alwassef 2016	38.31	12.83	41	66.27	15.7	49	3.5%	−1.92 [−2.42, −1.41]
Deye 2020	25.5	2.67	109	29.75	3.17	221	3.7%	−1.41 [−1.66, −1.15]
Kim 2020	53.8	35.3	19	199.3	144.7	64	3.4%	−1.12 [−1.66, −0.58]
Larsson 2014	19.74	6.29	57	22.69	7.11	68	3.6%	−0.43 [−0.79, −0.08]
Martinez-Losas 2020	37.33	5.18	174	47.6	5.77	146	3.7%	−1.88 [−2.14, −1.61]
Mörtberg 2011	2.31	1.4	14	3.17	0.8	17	3.2%	−0.75 [−1.49, −0.02]
Petermichl 2021	26	1.7	17	27.94	2.4	46	3.4%	−0.86 [−1.43, −0.28]
Reisinger 2007	15.73	1.48	90	19.33	3.99	87	3.7%	−1.20 [−1.52, −0.88]
Rundgren 2009	13.9	2.45	56	14.5	1.93	46	3.6%	−0.27 [−0.66, 0.12]
Shin 2023	32.25	4.3	69	38.38	3.88	185	3.7%	−1.53 [−1.84, −1.22]
Shinozaki 2009	25.48	11.33	13	35.36	13.35	67	3.4%	−0.75 [−1.36, −0.14]
Storm 2012	24.23	5.9	11	32.92	7.24	24	3.1%	−1.24 [−2.01, −0.46]
**Subtotal (95% CI)**			**670**			**1020**	**41.9%**	**−1.13 [−1.45, −0.80]**
Heterogeneity: $Tau^2 = 0.27$; $Chi^2 = 83.81$, df = 11 (P < 0.00001); $I^2 = 87\%$								
Test for overall effect: Z = 6.85 (P < 0.00001)								
**Total (95% CI)**			**1383**			**1975**	**100.0%**	**−1.26 [−1.59, −0.93]**
Heterogeneity: $Tau^2 = 0.76$; $Chi^2 = 442.90$, df = 28 (P < 0.00001); $I^2 = 94\%$								
Test for overall effect: Z = 7.43 (P < 0.00001)								
Test for subgroup differences: $Chi^2 = 0.46$, df = 1 (P = 0.50), $I^2 = 0\%$								

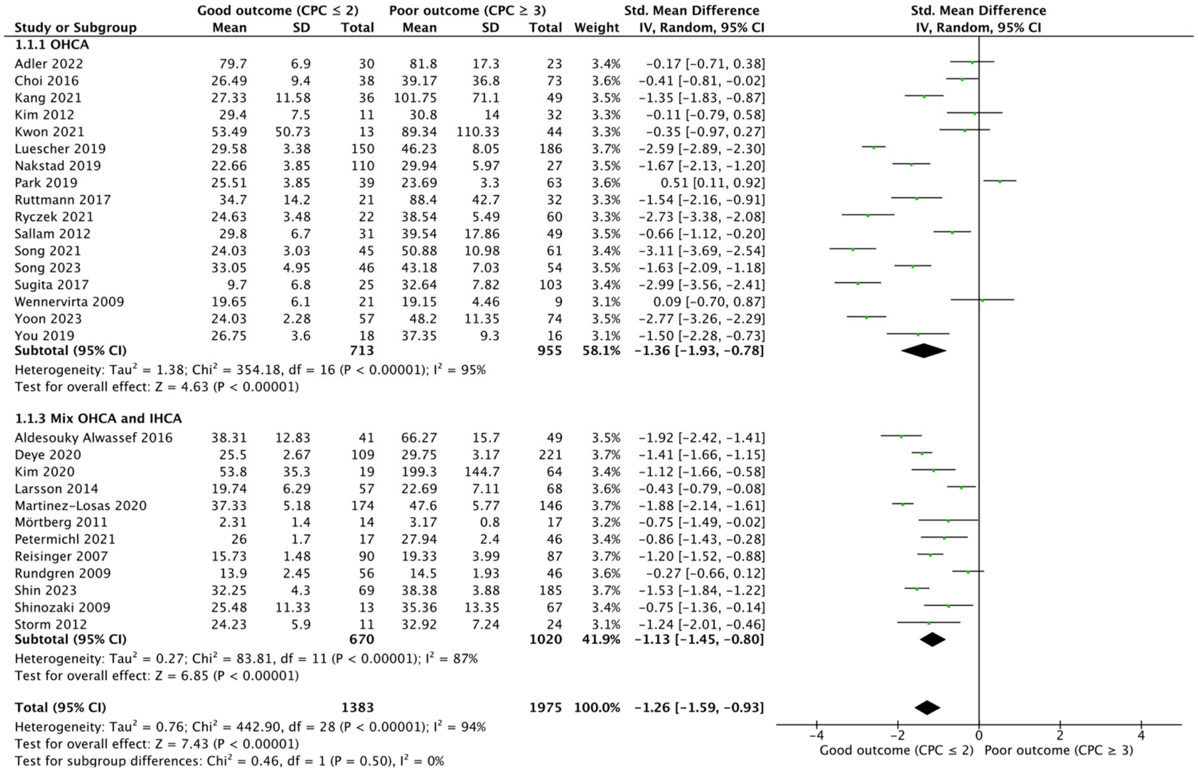

Figure S2: Forest plot of NSE levels among good vs. poor neurological outcome measured at baseline.

Study or Subgroup	Good outcome (CPC ≤ 2) Mean	SD	Total	Poor outcome (CPC ≥ 3) Mean	SD	Total	Weight	Std. Mean Difference IV, Random, 95% CI
**1.2.1 OHCA**								
Adler 2022	74.1	11.3	30	86.8	15.7	23	2.9%	−0.94 [−1.51, −0.36]
Choi 2016	23.95	11	35	53.87	39.3	61	2.9%	−0.92 [−1.36, −0.49]
Kang 2021	37.63	18.13	36	121.75	70.85	49	2.9%	−1.51 [−2.00, −1.02]
Kwon 2021	35.47	20.63	13	164.1	144.84	44	2.8%	−0.99 [−1.63, −0.34]
Luescher 2019	27.75	3	150	61.98	13.45	186	3.0%	−3.35 [−3.68, −3.01]
Nakstad 2019	27.2	20.5	128	68.6	82.5	131	3.0%	−0.68 [−0.93, −0.43]
Park 2019	23.74	3.34	39	47.89	8.61	63	2.8%	−3.38 [−4.00, −2.76]
Rafecas 2020	27.08	2.75	96	47.6	6.17	69	2.8%	−4.53 [−5.12, −3.95]
Ryczek 2021	24.64	3.84	22	70.45	24.79	60	2.8%	−2.12 [−2.71, −1.53]
Ryoo 2020	37.4	4.2	62	104.45	24.47	98	2.9%	−3.45 [−3.95, −2.95]
Son 2020	26.23	3.48	25	54.1	15.05	32	2.8%	−2.38 [−3.08, −1.69]
Song 2021	27.55	3.8	40	118.98	60.83	55	2.9%	−1.95 [−2.45, −1.46]
Song 2023	24.05	4	46	56.1	12.35	54	2.8%	−3.36 [−3.97, −2.74]
Sugita 2017	13.21	4.04	25	34.81	6.49	103	2.8%	−3.52 [−4.14, −2.90]
Wennervirta 2009	22.65	7.85	21	30.23	12.53	9	2.7%	−0.78 [−1.59, 0.03]
You 2019	26.4	3.55	18	125.58	68.29	16	2.6%	−2.07 [−2.92, −1.22]
**Subtotal (95% CI)**			**786**			**1053**	**45.6%**	**−2.25 [−2.90, −1.60]**
Heterogeneity: Tau² = 1.66; Chi² = 394.25, df = 15 (P < 0.00001); I² = 96%								
Test for overall effect: Z = 6.79 (P < 0.00001)								
**1.2.3 Mix OHCA and IHCA**								
Daubin 2011	29.15	5.75	25	65.45	17.25	72	2.9%	−2.37 [−2.94, −1.80]
Deye 2020	25	2.67	109	50.25	10.83	221	3.0%	−2.80 [−3.11, −2.48]
Haertel 2023	33.5	22.4	25	79.1	57.6	165	2.9%	−0.84 [−1.26, −0.41]
Hasslacher 2014	30.25	4.75	65	38.75	7.25	69	3.0%	−1.37 [−1.75, −0.99]
Kim 2023	60.58	5.99	9	64.03	14.98	16	2.7%	−0.26 [−1.09, 0.56]
Larsson 2014	14.02	3.78	57	37.72	15.11	68	2.9%	−2.06 [−2.50, −1.62]
Lascarrou 2021	21.78	3.66	13	44.43	11.58	88	2.8%	−2.06 [−2.71, −1.41]
Martinez-Losas 2020	35.55	3.83	174	61.93	9.75	146	3.0%	−3.67 [−4.03, −3.31]
Mörtberg 2011	1.83	0.69	14	3.86	2.25	17	2.7%	−1.14 [−1.91, −0.37]
Ok 2021	42.33	2.09	20	51.96	5.31	34	2.8%	−2.15 [−2.84, −1.46]
Oksanen 2009	19.15	3.05	50	19.55	2.2	40	3.0%	−0.15 [−0.56, 0.27]
Petermichl 2021	35.6	3.9	17	125.14	35.99	46	2.7%	−2.85 [−3.61, −2.10]
Reisinger 2007	16.2	1.73	90	30.04	12.24	87	3.0%	−1.59 [−1.93, −1.25]
Rundgren 2009	14.63	1.93	56	26.84	8.3	46	2.9%	−2.11 [−2.60, −1.62]
Schoerkhuber 1999	14.9	8.1	28	26	37	28	2.9%	−0.41 [−0.94, 0.12]
Shinozaki 2009	29.92	12.88	13	62.36	19.38	67	2.8%	−1.73 [−2.39, −1.08]
Storm 2012	21.36	2.78	11	51.71	18.61	24	2.6%	−1.90 [−2.76, −1.04]
Wihersaari 2022	9	1.23	128	15.28	3.35	120	3.0%	−2.51 [−2.85, −2.18]
Zellner 2013	24.32	3.79	61	40.41	12.29	62	2.9%	−1.75 [−2.17, −1.33]
**Subtotal (95% CI)**			**965**			**1416**	**54.4%**	**−1.78 [−2.23, −1.34]**
Heterogeneity: Tau² = 0.89; Chi² = 300.31, df = 18 (P < 0.00001); I² = 94%								
Test for overall effect: Z = 7.87 (P < 0.00001)								
**Total (95% CI)**			**1751**			**2469**	**100.0%**	**−1.99 [−2.36, −1.62]**
Heterogeneity: Tau² = 1.16; Chi² = 696.23, df = 34 (P < 0.00001); I² = 95%								
Test for overall effect: Z = 10.56 (P < 0.00001)								
Test for subgroup differences: Chi² = 1.34, df = 1 (P = 0.25), I² = 25.3%								

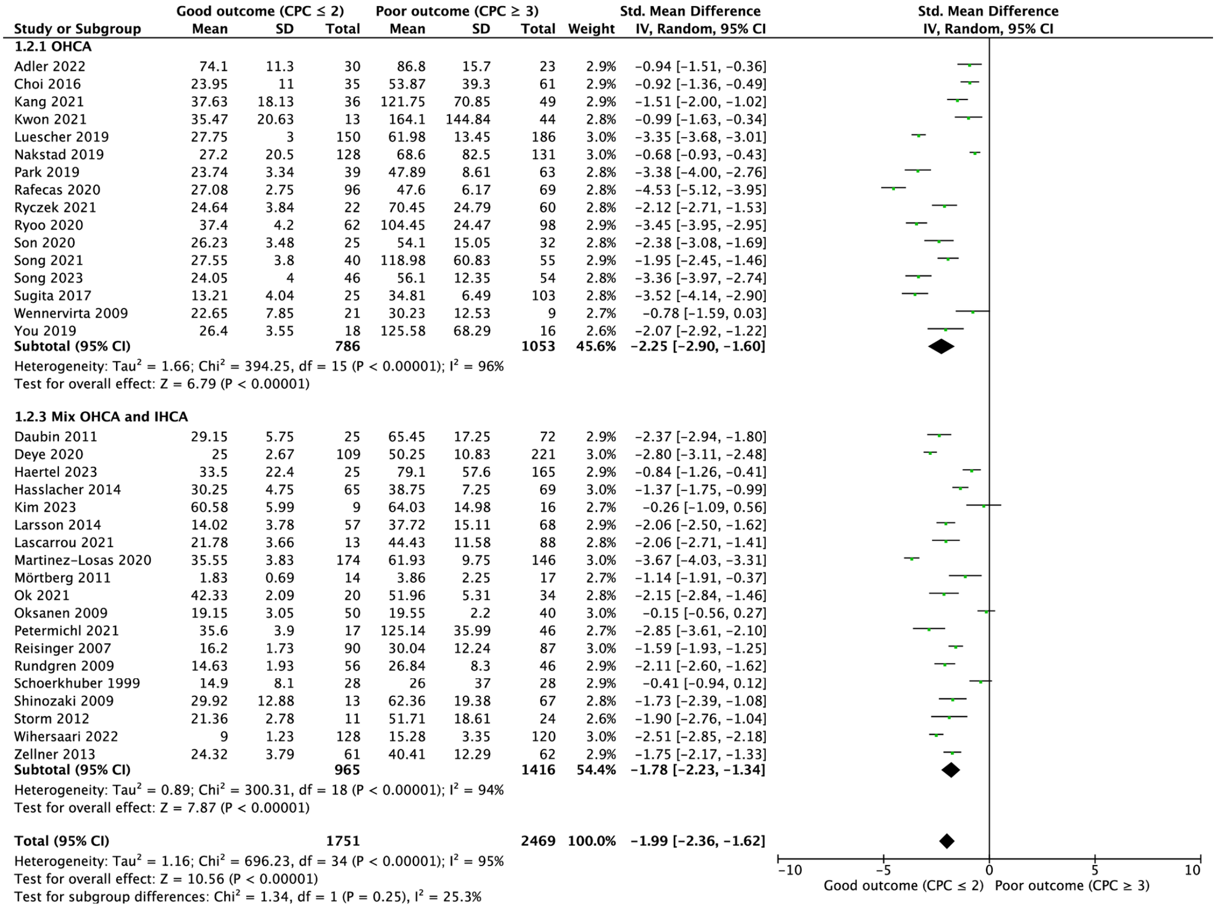

Figure S3: Forest plot of NSE levels among good vs. poor neurological outcome measured one day after cardiopulmonary resuscitation.

Study or Subgroup	Good outcome (CPC ≤ 2) Mean	SD	Total	Poor outcome (CPC ≥ 3) Mean	SD	Total	Weight	Std. Mean Difference IV, Random, 95% CI
**1.3.1 OHCA**								
Adler 2022	69.3	10.7	30	87.5	21.8	23	2.5%	−1.09 [−1.68, −0.51]
Ahn 2022	21.1	2.55	46	136.7	60.95	60	2.5%	−2.50 [−3.01, −1.98]
Benghanem 2022	30.25	5.5	18	119.75	48.5	64	2.5%	−2.06 [−2.67, −1.44]
Choi 2016	21.71	31.4	19	84.31	49.6	44	2.5%	−1.37 [−1.97, −0.78]
Jakkula 2019	18.35	1.93	78	47.2	12.2	40	2.5%	−3.96 [−4.59, −3.32]
Kaneko 2017	17	7.05	20	204	54.2	23	2.2%	−4.59 [−5.77, −3.41]
Kang 2021	29.43	13.73	36	126.23	11	49	2.1%	−7.85 [−9.13, −6.56]
Kim 2012	33.4	11.4	11	195.1	24.1	32	1.8%	−7.31 [−9.08, −5.55]
Lee 2021	22.5	3	171	119.75	27.83	304	2.6%	−4.34 [−4.68, −4.01]
Lee 2023	24.58	3.08	55	114.2	53.4	77	2.5%	−2.18 [−2.62, −1.74]
Luescher 2019	22.5	1.93	150	113.88	30.48	186	2.6%	−4.01 [−4.39, −3.64]
Nakstad 2019	21.2	14.7	128	116.8	176.7	131	2.6%	−0.76 [−1.01, −0.50]
Park 2019	18.75	2.9	39	73.83	16.79	63	2.4%	−4.10 [−4.80, −3.40]
Rafecas 2020	18.48	2.35	96	70.95	18.33	69	2.5%	−4.36 [−4.93, −3.79]
Ryczek 2021	23.6	4.23	22	109.47	36.02	60	2.5%	−2.74 [−3.39, −2.09]
Ryoo 2020	30.25	3.8	62	172.78	46.28	98	2.5%	−3.90 [−4.44, −3.37]
Song 2021	21.43	2.03	39	133.3	63.55	49	2.5%	−2.33 [−2.88, −1.79]
Song 2023	19.3	3.35	46	46.98	7.53	54	2.4%	−4.59 [−5.35, −3.83]
Sugita 2017	8.94	0.53	25	57.52	15.54	103	2.5%	−3.45 [−4.07, −2.84]
Wennervirta 2009	17.3	5.35	21	34.68	14.53	9	2.3%	−1.88 [−2.81, −0.95]
You 2019	21.33	2.08	18	165.25	61	16	2.2%	−3.36 [−4.45, −2.28]
**Subtotal (95% CI)**			**1130**			**1554**	**50.6%**	**−3.39 [−4.08, −2.71]**
Heterogeneity: Tau² = 2.41; Chi² = 641.91, df = 20 (P < 0.00001); I² = 97%								
Test for overall effect: Z = 9.71 (P < 0.00001)								
**1.3.2 Mix OHCA and IHCA**								
Aldesouky Alwassef 2016	44.37	15.44	41	82.49	18.19	49	2.5%	−2.22 [−2.76, −1.69]
Deye 2020	21.75	1.83	109	71.25	19.83	221	2.6%	−3.03 [−3.36, −2.71]
Haertel 2023	27.5	12.8	25	98.4	87.5	165	2.5%	−0.86 [−1.29, −0.43]
Helwig 2017	15.95	2.7	39	31.05	12.5	61	2.5%	−1.51 [−1.96, −1.05]
Kim 2023	47.85	12.28	9	256.75	85.83	16	2.2%	−2.90 [−4.10, −1.70]
Larsson 2014	11.39	3.19	57	39.35	18.27	68	2.5%	−2.04 [−2.47, −1.60]
Lascarrou 2021	19.4	5.21	13	95.43	30.85	88	2.4%	−2.60 [−3.29, −1.92]
Leão 2015	23.33	17.75	12	103.36	75.8	55	2.5%	−1.14 [−1.79, −0.48]
Martinez-Losas 2020	28.58	3.08	174	105.65	23.8	146	2.5%	−4.74 [−5.17, −4.31]
Mörtberg 2011	2.58	1.14	14	7.35	2.35	17	2.3%	−2.44 [−3.40, −1.48]
Oksanen 2009	15.4	1.8	50	23.88	5.73	40	2.5%	−2.08 [−2.60, −1.56]
Petermichl 2021	30.6	3.5	17	211.71	63.42	46	2.4%	−3.28 [−4.09, −2.47]
Reisinger 2007	11.65	1.53	90	70.92	49.42	87	2.6%	−1.70 [−2.05, −1.36]
Rossetti 2012	23.65	10.75	28	92.73	69.98	33	2.5%	−1.31 [−1.87, −0.75]
Rundgren 2009	12.68	1.73	56	40.46	15.76	46	2.5%	−2.59 [−3.12, −2.06]
Schoerkhuber 1999	11.9	5.7	28	37.6	58.2	28	2.5%	−0.61 [−1.15, −0.08]
Shinozaki 2009	28.82	13.02	13	85.59	17.12	67	2.4%	−3.40 [−4.20, −2.59]
Storm 2012	17.68	2.77	11	85.21	31.77	24	2.3%	−2.48 [−3.43, −1.54]
Wihersaari 2022	8.58	1.05	128	26.38	8.08	120	2.6%	−3.13 [−3.50, −2.76]
Zellner 2013	21.48	3.06	61	79.93	21.98	62	2.5%	−3.69 [−4.27, −3.10]
**Subtotal (95% CI)**			**975**			**1439**	**49.4%**	**−2.38 [−2.86, −1.89]**
Heterogeneity: Tau² = 1.13; Chi² = 328.41, df = 19 (P < 0.00001); I² = 94%								
Test for overall effect: Z = 9.57 (P < 0.00001)								
**Total (95% CI)**			**2105**			**2993**	**100.0%**	**−2.88 [−3.30, −2.46]**
Heterogeneity: Tau² = 1.72; Chi² = 993.81, df = 40 (P < 0.00001); I² = 96%								
Test for overall effect: Z = 13.58 (P < 0.00001)								
Test for subgroup differences: Chi² = 5.60, df = 1 (P = 0.02), I² = 82.1%								

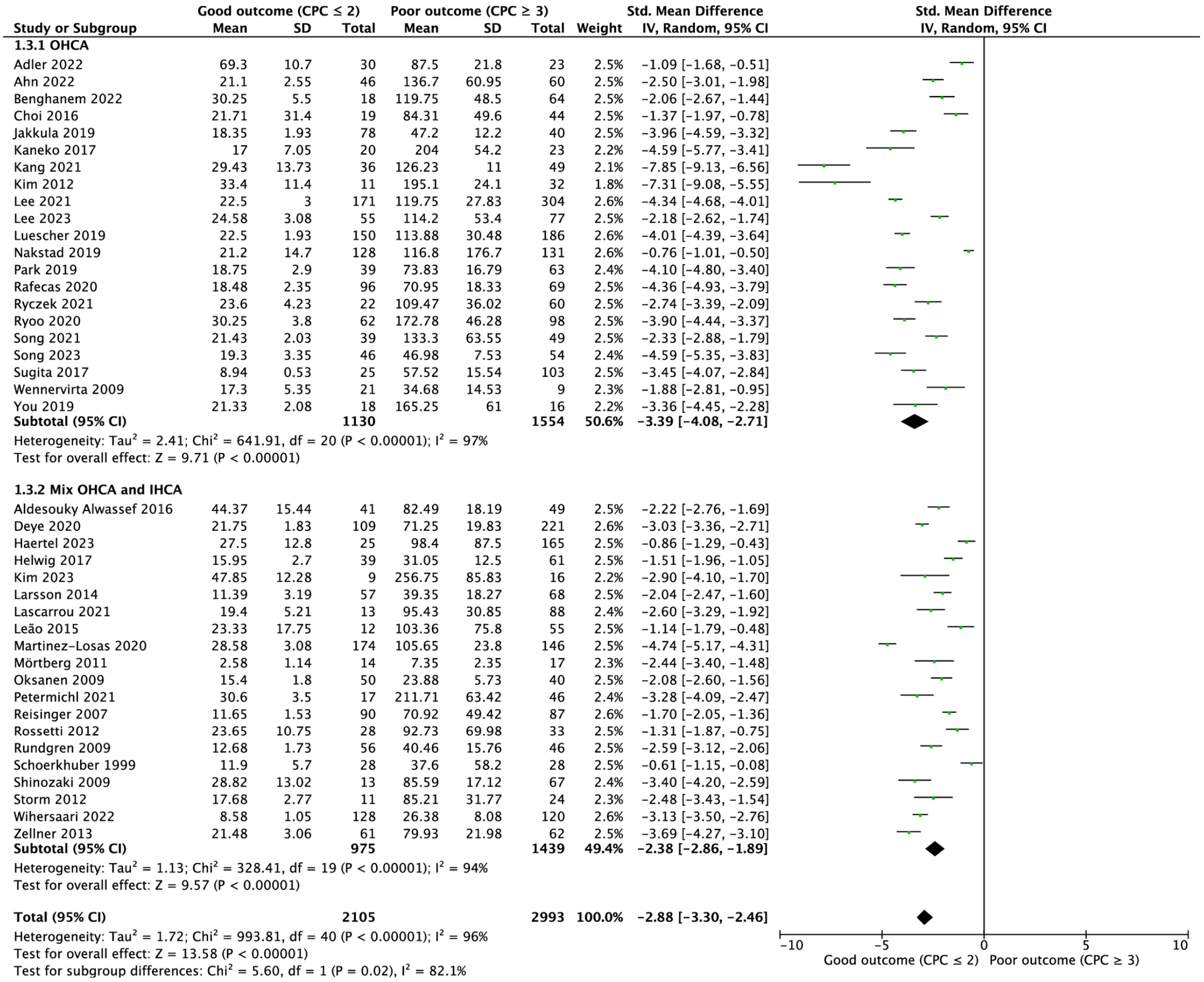

Figure S4: Forest plot of NSE levels among good vs. poor neurological outcome measured two days after cardiopulmonary resuscitation.

Study or Subgroup	Good outcome (CPC ≤ 2) Mean	SD	Total	Poor outcome (CPC ≥ 3) Mean	SD	Total	Weight	Std. Mean Difference IV, Random, 95% CI
**1.4.1 OHCA**								
Ahn 2022	19.35	3.35	46	138.93	59.23	60	2.6%	−2.66 [−3.19, −2.13]
Akin 2021	21.75	2.17	102	50.25	12.5	149	2.6%	−2.92 [−3.28, −2.56]
Akin 2022	28	3	59	115	13	158	2.5%	−7.73 [−8.52, −6.94]
Benghanem 2022	23.5	5	18	180.25	74.75	64	2.5%	−2.34 [−2.98, −1.70]
Chong 2020	23.55	5.6	20	189.5	47.5	25	2.3%	−4.57 [−5.72, −3.42]
Kang 2021	47	34	36	134.8	71.9	49	2.6%	−1.48 [−1.96, −0.99]
Kim 2012	30.7	10.9	11	238.1	161.7	32	2.5%	−1.45 [−2.20, −0.69]
Kwon 2021	18.28	9.16	13	204.81	142.77	44	2.5%	−1.46 [−2.13, −0.78]
Lee 2019	32	15.3	20	149.9	129.2	13	2.5%	−1.42 [−2.20, −0.63]
Lee 2021	18.75	2.5	171	157	40	304	2.6%	−4.31 [−4.64, −3.97]
Luescher 2019	19.7	1.97	150	118.6	29.28	186	2.6%	−4.52 [−4.93, −4.11]
Maher 2023	23	4.75	23	113.63	27.38	36	2.4%	−4.13 [−5.06, −3.20]
Nakstad 2019	14.95	4.69	110	105.5	47.15	27	2.5%	−4.26 [−4.93, −3.60]
Park 2019	17.07	2.08	39	84.3	23.38	63	2.5%	−3.62 [−4.26, −2.97]
Roger 2015	14.4	7.4	27	158.5	163.7	53	2.6%	−1.07 [−1.56, −0.57]
Ryczek 2021	19.47	3.58	22	156.5	50	60	2.5%	−3.16 [−3.85, −2.46]
Ryoo 2020	29	4.17	62	174.15	49.07	98	2.6%	−3.75 [−4.27, −3.23]
Song 2021	19.73	3.58	38	135	59.7	45	2.5%	−2.59 [−3.18, −2.00]
Song 2023	17.5	3.1	46	64.25	28.4	54	2.6%	−2.21 [−2.71, −1.71]
Stammet 2013	18.19	2.77	41	65.42	15.56	34	2.4%	−4.38 [−5.24, −3.53]
Stefanizzi 2022	37.9	19.4	286	203.78	129.82	256	2.7%	−1.83 [−2.04, −1.63]
Wurm 2022	28.33	8.08	21	39.78	16.58	49	2.6%	−0.78 [−1.30, −0.25]
You 2019	17.8	1.7	18	188.95	64.3	16	2.2%	−3.79 [−4.97, −2.62]
**Subtotal (95% CI)**			**1379**			**1875**	**57.7%**	**−3.04 [−3.62, −2.46]**
Heterogeneity: Tau² = 1.90; Chi² = 634.09, df = 22 (P < 0.00001); I² = 97%								
Test for overall effect: Z = 10.27 (P < 0.00001)								
**1.4.2 Mix OHCA and IHCA**								
Chung-Esaki 2018	15.7	3.2	42	36.98	2.88	56	2.3%	−6.99 [−8.07, −5.91]
Daubin 2011	15.6	1.75	25	136.5	51.75	72	2.5%	−2.68 [−3.28, −2.08]
Haertel 2023	25.9	15.1	25	87.5	79.7	165	2.6%	−0.82 [−1.25, −0.39]
Hasper 2009	18.3	2.8	69	89.5	43.5	102	2.6%	−2.10 [−2.48, −1.73]
Hasslacher 2014	19.5	1.5	65	96.25	37.75	69	2.6%	−2.81 [−3.30, −2.33]
Hermann 2023	28.75	5.75	47	184.5	48	134	2.6%	−3.74 [−4.25, −3.23]
Kim 2023	35.03	11.74	9	231.33	85.07	16	2.2%	−2.75 [−3.91, −1.58]
Kirsch 2021	20.3	2.8	22	139.38	13.78	69	2.0%	−9.74 [−11.27, −8.22]
Larsson 2014	8.4	0.99	57	50.29	25.96	68	2.6%	−2.17 [−2.62, −1.73]
Lascarrou 2021	10.68	3.62	13	105	35.53	88	2.5%	−2.81 [−3.51, −2.10]
Martinez-Losas 2020	21.35	2.57	174	133.2	31.57	146	2.6%	−5.21 [−5.68, −4.75]
Petermichl 2021	20.4	2.2	17	179.32	54.34	46	2.4%	−3.36 [−4.18, −2.54]
Reisinger 2007	12.08	1.88	90	63.91	43.77	85	2.6%	−1.69 [−2.04, −1.34]
Rundgren 2009	11.88	1.33	56	38.33	15.78	46	2.6%	−2.47 [−2.99, −1.95]
Schefold 2009	20.75	3.25	44	123.65	50.8	28	2.5%	−3.22 [−3.93, −2.50]
Schoerkhuber 1999	9.6	2.9	28	32	27.4	28	2.6%	−1.13 [−1.70, −0.57]
Storm 2012	16.78	5.82	11	84.35	48.81	24	2.4%	−1.62 [−2.43, −0.80]
**Subtotal (95% CI)**			**794**			**1242**	**42.3%**	**−3.16 [−3.86, −2.45]**
Heterogeneity: Tau² = 2.07; Chi² = 431.30, df = 16 (P < 0.00001); I² = 96%								
Test for overall effect: Z = 8.78 (P < 0.00001)								
**Total (95% CI)**			**2173**			**3117**	**100.0%**	**−3.09 [−3.52, −2.65]**
Heterogeneity: Tau² = 1.87; Chi² = 1068.45, df = 39 (P < 0.00001); I² = 96%								
Test for overall effect: Z = 13.81 (P < 0.00001)								
Test for subgroup differences: Chi² = 0.07, df = 1 (P = 0.80), I² = 0%								

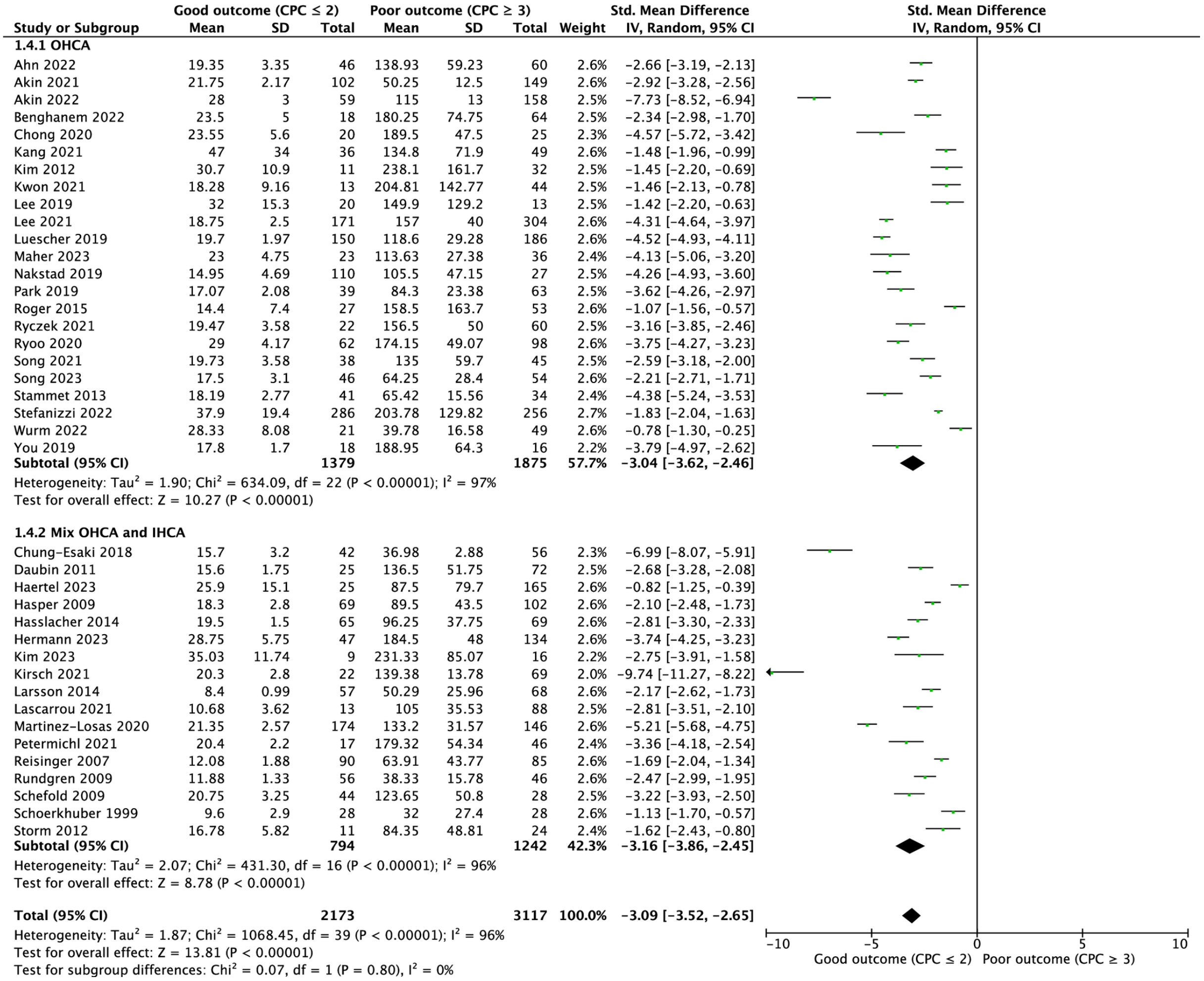

Figure S5: Forest plot of NSE levels among good vs. poor neurological outcome measured three days after cardiopulmonary resuscitation.

Study or Subgroup	Good outcome (CPC ≤ 2) Mean	SD	Total	Poor outcome (CPC ≥ 3) Mean	SD	Total	Weight	Std. Mean Difference IV, Random, 95% CI
**1.5.1 OHCA**								
Andersson 2022	28.25	2.5	175	117	28	374	12.8%	−3.83 [−4.12, −3.54]
Kim 2020b	24.53	5.03	34	111.6	32.4	75	12.4%	−3.19 [−3.78, −2.60]
Nakstad 2019	31.9	20.1	128	131.3	182.5	131	12.9%	−0.76 [−1.01, −0.51]
**Subtotal (95% CI)**			**337**			**580**	**38.1%**	**−2.59 [−4.81, −0.37]**
Heterogeneity: $Tau^2 = 3.80$; $Chi^2 = 257.21$, df = 2 (P < 0.00001); $I^2 = 99\%$								
Test for overall effect: Z = 2.29 (P = 0.02)								
**1.5.2 IHCA**								
Andersson 2022	24.5	3.33	98	64	19.67	146	12.8%	−2.56 [−2.91, −2.22]
Barbella 2020	27.85	8.67	9	204.13	163.18	62	12.1%	−1.14 [−1.86, −0.41]
**Subtotal (95% CI)**			**107**			**208**	**24.9%**	**−1.89 [−3.28, −0.49]**
Heterogeneity: $Tau^2 = 0.93$; $Chi^2 = 12.13$, df = 1 (P = 0.0005); $I^2 = 92\%$								
Test for overall effect: Z = 2.65 (P = 0.008)								
**1.5.3 Mix OHCA and IHCA**								
Daubin 2011	29.4	5.5	25	99.3	33.5	72	12.4%	−2.38 [−2.95, −1.81]
Schoerkhuber 1999	16.5	7.2	28	45.2	57.3	28	12.5%	−0.69 [−1.23, −0.15]
Wolff 2009	19.7	2.5	28	83.58	37.93	21	12.0%	−2.53 [−3.30, −1.76]
**Subtotal (95% CI)**			**81**			**121**	**37.0%**	**−1.85 [−3.07, −0.63]**
Heterogeneity: $Tau^2 = 1.06$; $Chi^2 = 23.36$, df = 2 (P < 0.00001); $I^2 = 91\%$								
Test for overall effect: Z = 2.98 (P = 0.003)								
**Total (95% CI)**			**525**			**909**	**100.0%**	**−2.14 [−3.13, −1.15]**
Heterogeneity: $Tau^2 = 1.96$; $Chi^2 = 299.84$, df = 7 (P < 0.00001); $I^2 = 98\%$								
Test for overall effect: Z = 4.24 (P < 0.0001)								
Test for subgroup differences: $Chi^2 = 0.35$, df = 2 (P = 0.84), $I^2 = 0\%$								

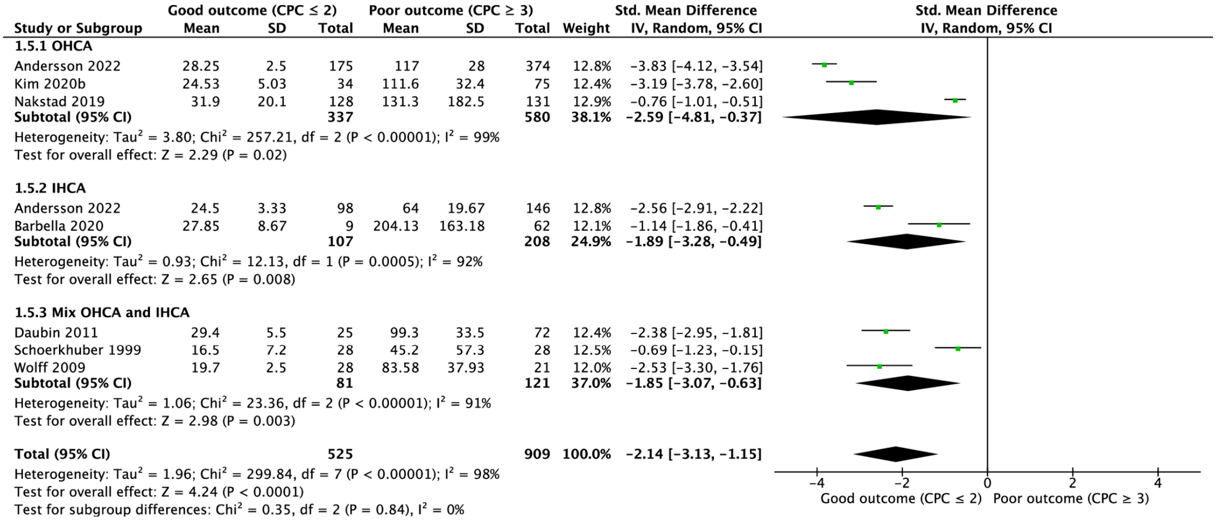

Figure S6: Forest plot of peak of NSE levels among good vs. poor neurological outcome groups.
